# DDR1 Drives Collagen Remodeling and Immune Exclusion: Pan-Cancer Insights and Therapeutic Targeting in Pancreatic Ductal Adenocarcinoma

**DOI:** 10.3390/ijms26167731

**Published:** 2025-08-10

**Authors:** Xuan Huang, Guangjun Jing, Kudelaidi Kuerban, Jiajun Fan, Mei Yu, Shanglin Yang, Wei Chen, Litao Huang, Dianwen Ju, Yi Zhun Zhu, Li Ye

**Affiliations:** 1 Department of Biological Medicines & Shanghai Engineering Research Center of Immunotherapeutics, School of Pharmaceutical Sciences, Fudan University, Shanghai 201100, China; 21111030066@m.fudan.edu.cn (X.H.); 21111030008@m.fudan.edu.cn (G.J.); kudelaidi@fudan.edu.cn (K.K.); jiajunfan12@fudan.edu.cn (J.F.); dianwenju@fudan.edu.cn (D.J.); 2 School of Pharmacy & Laboratory of Drug Discovery from Natural Resources and Industrialization, Macau University of Science and Technology, Macau SAR, China; ymuabelinda@163.com (M.Y.); 2230028448@student.must.edu.mo (S.Y.); 3230005606@student.must.edu.mo (W.C.); lihuang@must.edu.mo (L.H.);; 3 School of Life Science and Technologies, Tongji University, Shanghai 200065, China

**Keywords:** discoidin domain receptor 1, extracellular matrix, pan-cancer analyses, immune infiltration, pancreatic ductal adenocarcinoma

## Abstract

Discoidin domain receptor 1 (DDR1), a collagen-binding receptor tyrosine kinase, plays a key role in extracellular matrix remodeling, tumor progression, and immune evasion. However, DDR1’s comprehensive role across diverse cancers and its therapeutic potential in immune-resistant tumors remain poorly defined. We performed a pan-cancer analysis integrating bulk transcriptomic datasets, single-cell RNA sequencing, and pathway enrichment to evaluate *DDR1* expression, genetic alterations, and its associations with immune cell infiltration and clinical outcomes. *DDR1* was consistently overexpressed in 21 cancer types, correlating with poor prognosis and reduced immune cell infiltration. Mechanistically, DDR1 promoted collagen remodeling, immune exclusion, and upregulated immunosuppressive pathways. Single-cell analysis in pancreatic ductal adenocarcinoma (PDAC) revealed *DDR1*-high ductal cells associated with reduced cytotoxic T cell infiltration and increased regulatory T cell populations. Therapeutic blockade of DDR1 in an immunocompetent KPC mouse model of PDAC disrupted collagen architecture, enhanced CD8^+^ T cell infiltration, and improved responses to chemotherapy, highlighting a direct link between DDR1 inhibition and immune reactivation. These findings establish DDR1 as a key mediator of collagen-driven immune resistance and a promising therapeutic target for overcoming immune exclusion, especially in PDAC and other collagen-rich solid tumors.

## 1. Introduction

Discoidin domain receptor 1 (DDR1) is a collagen-binding receptor tyrosine kinase (RTK) that preserves extracellular matrix (ECM) homeostasis and, consequently, influences tissue repair, fibrosis, and tumor progression [1]. Mounting evidence also identifies DDR1 as an oncogenic driver in a variety of malignancies. In our previous work, *DDR1* was markedly overexpressed in colorectal and gastric cancers, where it predicted poor survival and low immune cell infiltration, underscoring its multifaceted contribution to tumor biology [2].

Mechanistically, DDR1 activation reorganizes the ECM, a prerequisite for tumor expansion and dissemination. In breast cancer, stromal DDR1 promotes collagen deposition and tumor cell motility, fueling aggressive phenotypes [3]. DDR1 further potentiates invasion by inducing IL-6 secretion in adipose stromal cells [4] and by stimulating CXCL5 production through the DDR1/PKCθ/SYK/NF-κB axis; the resulting neutrophil extracellular trap formation and regulatory T cell (Treg) recruitment accelerate metastasis [5,6]. Downstream, CXCL5-triggered PI3K/AKT signaling and DDR1-dependent JNK activation converge to elevate PD-L1 expression [7], thereby reinforcing an immunosuppressive tumor microenvironment (TME) [8].

Beyond these actions, DDR1 directly impedes antitumor immunity. In colorectal cancer it suppresses IL-18 production and curtails CD4^+^/CD8^+^ T cell infiltration [7]. Its extracellular domain (ECD) binds collagen and actively realigns collagen fibers within the TME, a trait linked to T cell exclusion in triple-negative breast cancer (TNBC) [9,10,11]. Preclinical blockade of DDR1 restructures collagen, enhances CD8^+^ T cell penetration, and suppresses tumor growth [10,12]; monoclonal antibodies against the DDR1-ECD can even induce complete tumor regression while boosting interferon-γ (IFN-γ) production [10,13].

Despite these insights, most DDR1 research has centered on TNBC, leaving its broader immunomodulatory roles—particularly in tumors with complex stromal niches—poorly defined [14]. To address this gap, we performed a systematic pan-cancer analysis that interrogated *DDR1*’s diagnostic and prognostic value, genetic alterations, pathway associations, and immunotherapeutic relevance across multiple tumor types.

Pancreatic ductal adenocarcinoma (PDAC) emerged as a paradigmatic case of *DDR1* dysregulation. PDAC exemplifies the “immune-desert” phenotype: its dense desmoplastic stroma impedes drug penetration and sustains immune evasion, contributing to resistance against both immune checkpoint inhibitors (ICIs) and targeted agents [15]. Our pan-cancer screening revealed that PDAC shows concurrent *DDR1* over-expression, low immune/stromal scores, and dismal prognosis—features aligned with *DDR1*’s known roles in collagen remodeling. Leveraging single-cell RNA sequencing (scRNA-seq) datasets, we delineated *DDR1*-centered stromal–immune crosstalk in PDAC.

To experimentally dissect the immune dependency and therapeutic potential of DDR1 blockade in PDAC, we employed two distinct murine models. For loss-of-function studies in an immunodeficient context, we used NOD.Cg-*Prkdc^scid^Il2rg^tm1Wjl^*/SzJ strain (NOD scid gamma, NSG) mice, which lack functional T, B, and NK cells, allowing evaluation of tumor-intrinsic and stroma-intrinsic drug effects independent of adaptive immunity [16]. To more faithfully recapitulate the immunocompetent tumor microenvironment and enable assessment of immune-mediated antitumor responses, we established subcutaneous syngeneic tumors using the KPC-derived PDAC cell line (*LSL-Kras^G12D/+^; LSL-Trp53^R172H/+^; Pdx-1-Cre*, on *C57BL/6J* background) in *C57BL/6J* mice [16,17]. The KPC model closely mirrors key features of human PDAC, including robust desmoplasia, aggressive progression, and immune evasion, thus providing translational relevance for preclinical immunotherapeutic evaluation. Finally, a therapeutic DDR1 blockade in a KPC PDAC mouse model remodeled the collagen scaffold, restored antitumor immunity, and curtailed tumor growth [18].

Collectively, these findings position DDR1 as a central orchestrator of collagen-driven immune exclusion and as a promising biomarker and therapeutic target in collagen-rich tumors such as PDAC, thereby extending the potential of DDR1-directed therapies beyond TNBC.

## 2. Results

### 2.1. Expression of DDR1 in Pan-Cancer

**Figure 1:**
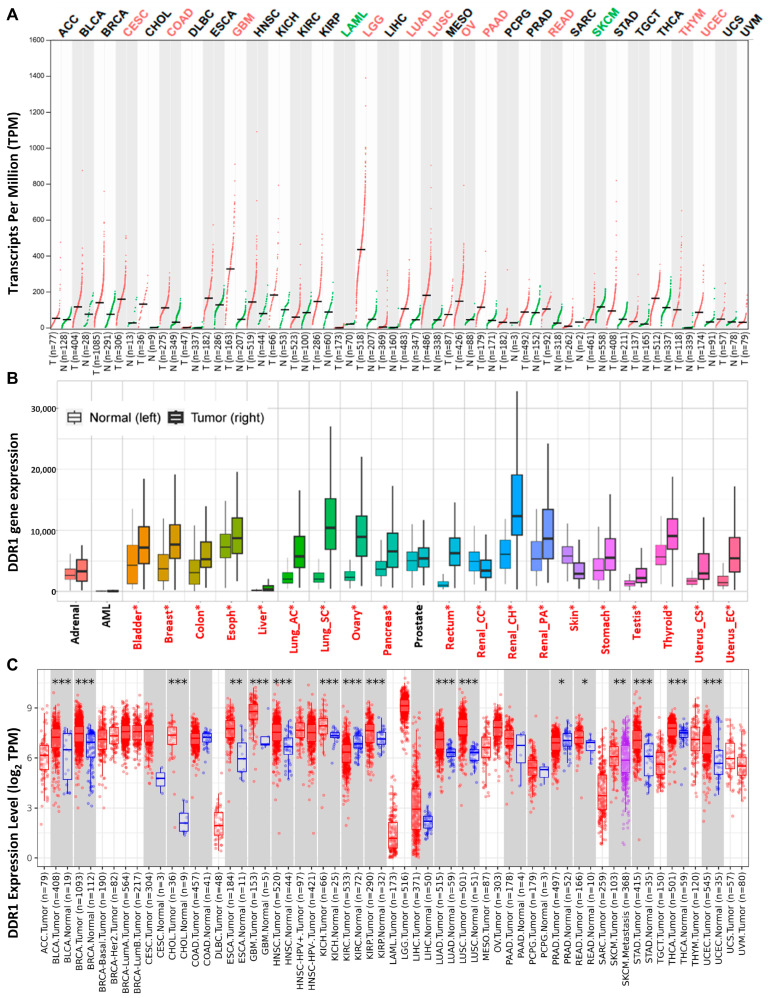
Pan-cancer analysis of *DDR1* expression. (**A**) Dot plot showing *DDR1* mRNA expression levels (measured in Transcripts Per Million, TPM) in tumor tissues (T, red dots) and paired normal tissues (N, green dots) across various cancer types using RNA-seq data from the GEPIA 2.0 database. Each dot represents a single sample. Cancer type abbreviations along the x-axis correspond to TCGA (The Cancer Genome Atlas) project codes. The colors of the cancer type labels indicate the statistical significance of *DDR1* expression differences between tumor and normal tissues. Red labels indicate cancer types where *DDR1* expression is significantly upregulated in tumor tissues compared to paired normal tissues. Green labels indicate cancer types where *DDR1* expression is significantly downregulated in tumor tissues compared to paired normal tissues. Black labels indicate cancer types with no statistically significant difference in *DDR1* expression between tumor and paired normal tissues. Statistical comparisons between tumor and normal groups were performed using one-way analysis of variance (ANOVA). (**B**) Bar plot of *DDR1* expression (gene chip) across normal and cancer tissues from the KM-Plotter database. Red asterisks (*) above tissue names indicate significant differences (Mann–Whitney * *p* < 0.05) with expression >10 in either tumor or normal tissues. (**C**) Box plots showing *DDR1* expression across all TCGA tumors in the TIMER 2.0 database. Red boxes represent tumor tissues; blue boxes denote paired adjacent normal tissues. Statistical significance from RNA-Seq differential analysis (edgeR) is annotated as: * *p* < 0.05; ** *p* < 0.01; *** *p* < 0.001.

A comprehensive analysis of *DDR1* expression patterns across diverse tissues and cellular subtypes was conducted utilizing the GTEx database and The Human Protein Atlas. The results demonstrated pronounced tissue-specific regulation, with *DDR1* exhibiting elevated expression in epithelial cells and glial cells while showing significantly reduced expression in neuronal cells, blood cells, and immune cell populations (Supplementary Figures S1 and S2). To further investigate *DDR1* expression patterns across cancers, we integrated data from three independent sources: GEPIA 2.0 (Figure 1A), KM-Plotter (Figure 1B), and TIMER 2.0 (Figure 1C). Each database is based on distinct sample collections and analytical platforms—for instance, GEPIA 2.0 predominantly utilizes RNA-seq data from TCGA and GTEx, while KM-Plotter incorporates a large volume of gene chip–based data.

Notably, *DDR1* expression patterns varied across the databases. For example, while KM-Plotter and TIMER 2.0 both reported high *DDR1* expression in invasive breast carcinoma (IBC, referred to as BRCA in TCGA), GEPIA 2.0 showed no significant upregulation compared to paired normal tissues. These discrepancies likely stem from differences in sample size, data source, and statistical methodology. To mitigate potential bias and ensure comprehensive coverage of tumor types potentially associated with *DDR1* upregulation, we compiled the union of cancer types identified as *DDR1*-high across the three databases for downstream analyses, including survival and immune correlation studies.

Based on this integrated approach, *DDR1* appears to be upregulated in several malignancies, including bladder urothelial carcinoma (BLCA), breast cancer (BRCA), cervical squamous cell carcinoma and endocervical adenocarcinoma (CESC), cholangiocarcinoma (CHOL), colon adenocarcinoma (COAD), esophageal carcinoma (ESCA), glioblastoma multiforme (GBM), head and neck squamous cell carcinoma (HNSC), kidney chromophobe (KICH), kidney renal clear cell carcinoma (KIRC), kidney renal papillary cell carcinoma (KIRP), brain lower grade glioma (LGG), lung adenocarcinoma (LUAD), lung squamous cell carcinoma (LUSC), ovarian serous cystadenocarcinoma (OV), pancreatic adenocarcinoma (PAAD), rectum adenocarcinoma (READ), stomach adenocarcinoma (STAD), thyroid carcinoma (THCA), uterine corpus endometrial carcinoma (UCEC), and thymoma (THYM).

**Figure 2:**
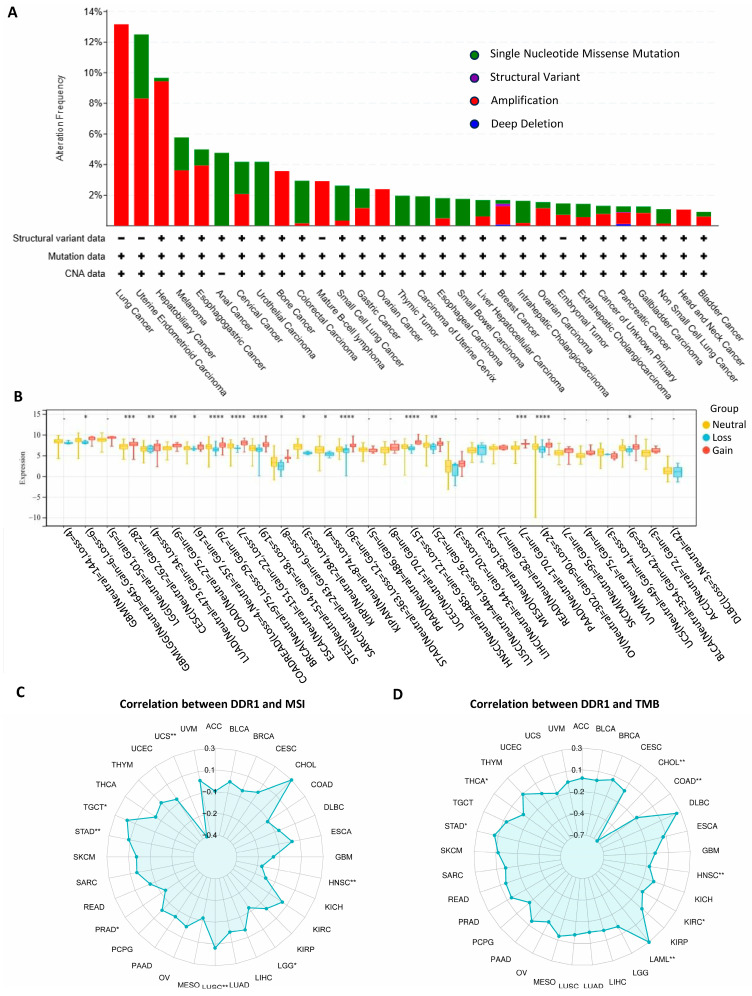
Genetic alterations, copy number variation, and genomic instability features associated with *DDR1* across cancers. (**A**) Alteration gene frequency of *DDR1* including single nucleotide missense mutation (green), structural variant (purple), gene amplification (red), and deletion (blue) in the various types of cancers using the cBioPortal. (**B**) Association between *DDR1* expression and copy number variation (CNV) states (neutral, loss, gain) across pan-cancer, presented as box plots. Statistical significance was assessed using the Wilcoxon rank-sum test for pairwise comparisons and the Kruskal–Wallis test for multiple groups (* *p* < 0.05; ** *p* < 0.01; *** *p* < 0.001; **** *p* < 0.0001). (**C**,**D**) Radar plots showing correlations between *DDR1* expression and (**C**) tumor mutational burden (TMB) or (**D**) microsatellite instability (MSI) across cancer types, assessed by Pearson’s correlation (* *p* < 0.05; ** *p* < 0.01).

The pan-cancer analysis of the *DDR1* genetic alterations is presented in Figure 2A. The frequency of genetic variation in *DDR1* was the highest in lung cancer (13%) and was mainly in the form of amplification. It shows that in patients with various cancer types, such as lung cancer, ovarian cancer, and PAAD, there is a significant occurrence of *DDR1* gene amplification. Our analysis revealed that copy number variations (CNVs) significantly influence *DDR1* expression among seventeen tumor types (Figure 2B) such as PAAD, OV, and STAD. These findings suggest that, in these cancers, CNV alterations may serve as a critical regulatory mechanism for *DDR1* expression, potentially impacting tumor biology and prognosis. A positive correlation was detected between *DDR1* and tumor mutational burden (TMB) in testicular germ cell tumors (TGCT), STAD, and LUSC (Figure 2C), and between *DDR1* and microsatellite instability (MSI) in STAD and acute myeloid leukemia (LAML) (Figure 2D).

### 2.2. Prognostic Value of DDR1 in Pan-Cancer

**Figure 3:**
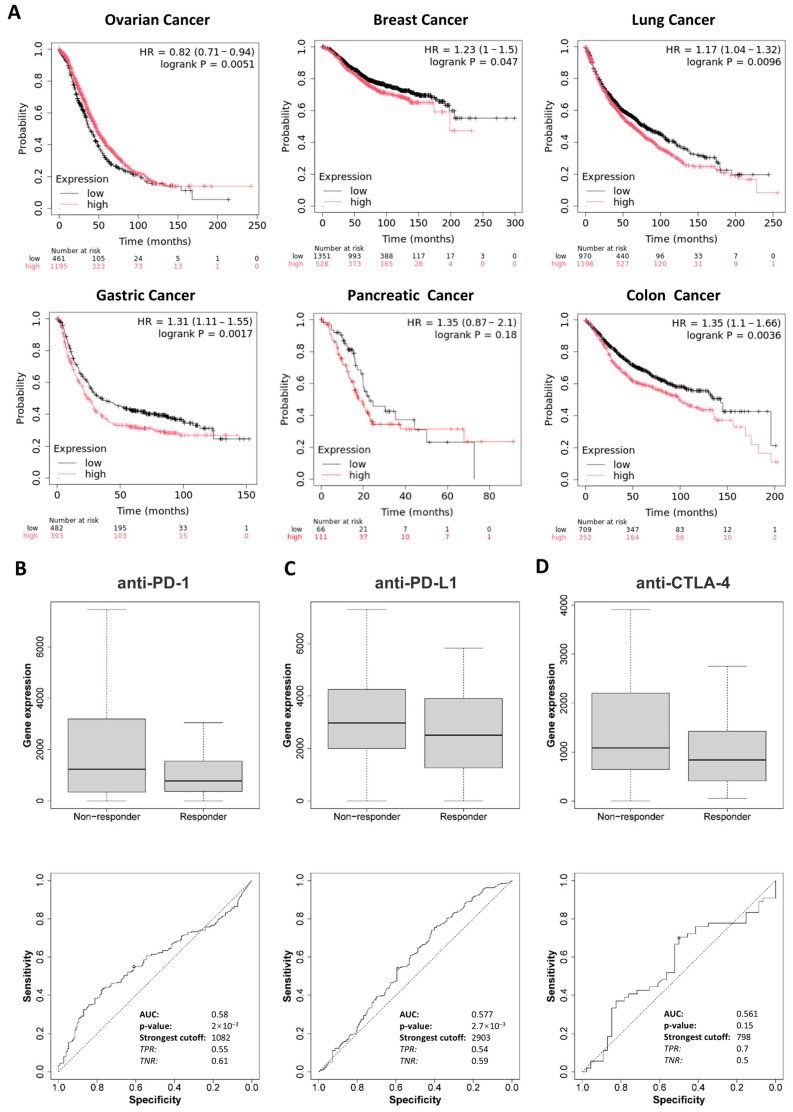
Prognosis value of *DDR1* from the KM plotter and ROC plotter. (**A**) Overall survival analysis of *DDR1* across cancers from the KM plotter. Red curves represent patients with high *DDR1* expression; black curves indicate patients with low *DDR1* expression. (**B**,**D**) The ROC plot of the association between *DDR1* expression and the response to anti-PD-1 (**B**), anti-PD-L1 (**C**), and anti-CTLA4 (**D**) therapy.

Kaplan–Meier survival analysis was conducted to evaluate the prognostic value of *DDR1* expression across ovarian, breast, lung, gastric, pancreatic, and colon cancer cohorts. High *DDR1* expression was significantly associated with poor overall survival (OS) in patients with breast, lung, and gastric cancers (Figure 3A). In contrast, elevated *DDR1* expression appeared to be associated with favorable prognosis in ovarian cancer. For PAAD, possibly due to limited sample size, no significant association was observed between *DDR1* expression and patient survival. These findings suggest that *DDR1* expression could serve as a detectable biomarker for prognostic prediction in these tumor types.

To further explore the clinical relevance of *DDR1*, we analyzed its relationship with immunotherapy response using the ROC plotter database, which includes patient cohorts treated with anti-PD-1 therapies (nivolumab and pembrolizumab), anti-PD-L1 therapies (atezolizumab and durvalumab), and anti-CTLA-4 therapy (ipilimumab) [19]. As shown in Figure 3B–D, high *DDR1* expression was significantly associated with weaker responses to anti-PD-1 and anti-PD-L1 treatments. Notably, this immunotherapy resistance may be partly explained by *DDR1*’s link to TMB and MSI, as positive correlations were observed between *DDR1* expression and TMB in STAD and LUSC, and with MSI in STAD and LAML (Figure 2C,D). Although TMB and MSI are typically predictive of favorable immunotherapy outcomes due to increased neoantigen load and immune activation [20,21], our findings suggest that DDR1 may counteract these benefits by fostering an immune-excluded tumor microenvironment. DDR1 could act downstream or independently of TMB/MSI to limit effector T cell infiltration or function, thereby blunting the therapeutic potential of checkpoint inhibitors. These observations underscore DDR1’s value as both a predictive biomarker and a therapeutic target, particularly in tumors where high immunogenicity fails to translate into effective immune responses.

**Figure 4:**
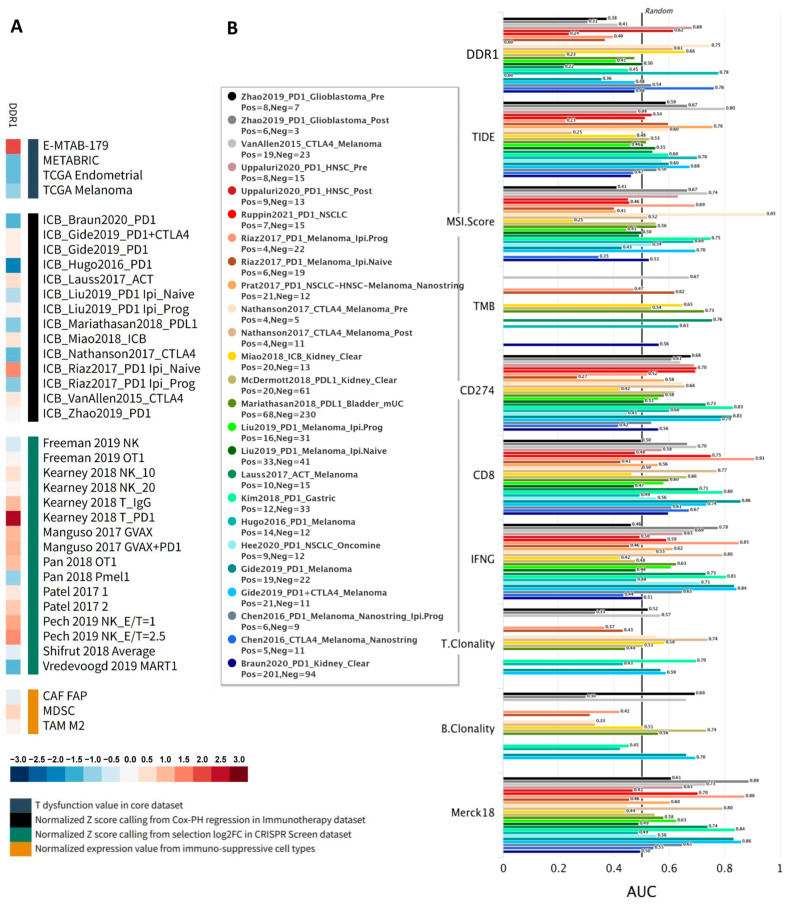
Pan-cancer prioritization and functional annotation identify *DDR1* as an immunosuppressive regulator and candidate biomarker for immune checkpoint blockade. (**A**) Prioritization of *DDR1* as a candidate immunotherapy target using the TIDE Gene-set module. The heat-map summarizes four immunosuppressive metrics, including T cell dysfunction, T cell exclusion, association with ICB survival, and CRISPR log-fold change, calculated across >50 datasets for the single candidate gene DDR1. The T dysfunction score shows how a gene interacts with cytotoxic T cells to influence patient survival outcome, and the T cell exclusion score assesses the gene expression levels in immunosuppressive cell types that drive T cell exclusion. The association score of (z-score in the Cox-PH regression) ICB survival outcome evaluates genes whose activities are correlated with ICB benefit. The normalized logFC in CRISPR screens help identify regulators whose knockout can mediate the efficacy of lymphocyte-mediated tumor killing in cancer models. (**B**) Evaluation of *DDR1* as a predictive biomarker for ICB response compared to nine standardized biomarkers. Horizontal bar plot summarizing the area-under-the-ROC-curve (AUC) obtained for the custom DDR1 signature and nine reference biomarkers: TIDE, MSI-score, tumor-mutational burden (TMB), *CD274* (PD-L1) expression, *CD8* signature, *IFNG* signature, T- or B-cell clonality scores, and Merk18 signature. Each colored bar corresponds to one of the 25 immune checkpoint-treated cohorts (color legend at left). Bar length reflects cohort-specific AUC.

We further explored the functional role of *DDR1* using the Gene Set Prioritization Module of the TIDE database, which integrates large-scale omics data and clinical cohorts, allowing cancer biologists to prioritize genes based on their clinical relevance and reproducibility across independent studies [22]. Specifically, this module evaluates associations of *DDR1* expression with immune checkpoint blockade (ICB) outcomes, T cell dysfunction and exclusion, and phenotypes from genetic screens across more than 50 cohorts. A heatmap summarizing these four immunosuppressive metrics—T cell dysfunction, T cell exclusion, association with ICB survival, and CRISPR log-fold change—revealed a robust immunosuppressive pattern for *DDR1* (Figure 4A). Red shading indicates positive correlations, highlighting that high *DDR1* expression is associated with increased T cell dysfunction, immune cell exclusion, and worse ICB survival outcomes. Conversely, blue shading represents negative CRISPR log-fold change scores, implying that *DDR1* knockout enhances lymphocyte-mediated tumor killing. This concordant pattern strongly supports *DDR1* as a clinically relevant target for combination immunotherapy.

Across all datasets analyzed, *DDR1* exhibited one of the widest dynamic ranges (standardized scores from approximately +2.63 to −2.05), suggesting strong context-dependent effects. Positive scores from CRISPR-based lymphocyte–tumor co-culture screens indicated that DDR1 disruption sensitizes tumor cells to cytotoxic T and NK cells, while adverse (positive) associations with ICB survival were consistently observed in several independent cohorts. Conversely, negative survival Z-scores in melanoma, renal, and TNBC cohorts indicate that low *DDR1* expression correlates with improved ICB outcomes, highlighting tumor-type specificity. Furthermore, cell-type–specific analyses showed *DDR1* enrichment within myeloid-derived suppressor cells (MDSC) and M2-polarized tumor-associated macrophages, aligning with its proposed role in fostering an immunosuppressive and exclusionary tumor microenvironment. Its previously established role in stromal and ECM remodeling further supports this immunomodulatory function.

Additionally, we compared *DDR1* against conventional immunotherapy biomarkers (TIDE, MSI Score, TMB, *CD274*, *CD8*, *IFNγ*, T clonality, B clonality, and Merck18) across 25 human immunotherapy cohorts to assess its predictive accuracy. *DDR1* achieved an area under the ROC curve (AUC) greater than 0.5 in 8 out of the 25 cohorts, outperforming established markers such as TMB, T clonality, and B clonality (Figure 4B), thereby demonstrating its promise as a single-gene predictor of ICB response. Collectively, these findings emphasize DDR1’s critical regulatory role in shaping the tumor immune microenvironment and support its potential application as a biomarker and therapeutic target in immunotherapy.

### 2.3. Functional Analysis of DDR1

**Figure 5:**
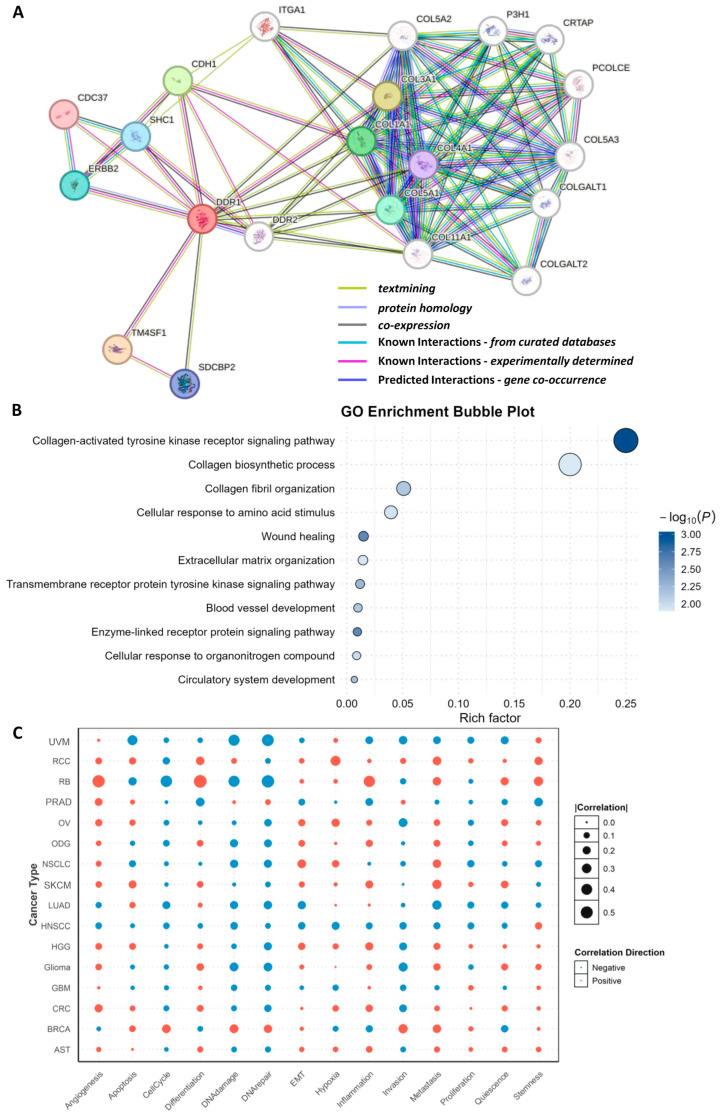
Multi-scale functional characterization of DDR1. (**A**) Protein–protein interaction (PPI) network centered on DDR1, constructed using the STRING database (v12.0). Edges represent high-confidence predicted associations. (**B**) Gene Ontology (GO) biological process enrichment analysis of DDR1-interacting proteins, also based on STRING-derived interactions. Top 10 enriched biological processes are grouped into four mechanistic categories. (**C**) Correlation of *DDR1* expression with 14 cancer-related functional states across 25 malignancies, derived from the CancerSEA database. Data represent single-cell transcriptomic associations between *DDR1* expression and defined cellular phenotypes such as proliferation, invasion, angiogenesis, and immune evasion.

Using the STING 12.0 database, we conducted a protein interaction analysis to predict DDR1-associated functional proteins (Figure 5A). The resulting network and functional modules revealed that the top predicted interactors of DDR1 include TM4SF1, COL3A1, CDH1, COL1A1, COL5A1, ERBB2, SHC1, SDCBP2, COL4A1, and CDC37. Notably, four of these interactors—COL3A1, COL1A1, COL5A1, and COL4A1—are members of the collagen family [23], underscoring DDR1’s central role in collagen-mediated signaling pathways. The interaction between DDR1 and various collagens suggests that DDR1 may contribute to tumor progression and metastasis by modulating ECM remodeling and dynamics. In addition, other predicted interactors such as TM4SF1, CDH1, and ERBB2 support DDR1’s involvement in key processes including cell adhesion, migration, and signal transduction. TM4SF1, a member of the transmembrane 4 superfamily, is known to facilitate cell motility and adhesion and has been implicated in the promotion of epithelial–mesenchymal transition (EMT), potentially enhancing DDR1’s pro-invasive function in cancer [24]. CDH1 (E-cadherin), a critical cell adhesion molecule, interacts with DDR1, further implicating DDR1 in cell–cell adhesion regulation and EMT-related processes [25]. ERBB2 (HER2), a receptor tyrosine kinase frequently dysregulated in breast and other cancers, also appears as a DDR1 interactor, suggesting a possible cooperative role between DDR1 and RTKs in promoting tumor cell proliferation and survival.

Following the identification of DDR1-interacting proteins, Gene Ontology (GO) enrichment analysis was performed. The ten most significantly enriched biological processes (BP) were categorized into four mechanistic groups (Figure 5B): (i) collagen-centric processes (collagen metabolic process and collagen fibril organization); (ii) ECM reorganization and wound healing (extracellular matrix organization, tissue remodeling, and wound healing); (iii) core signal transduction pathways (PI3K/Akt, MAPK, and JAK/STAT); (iv) cellular programs governing motility and stress adaptation (cell adhesion, cell migration, angiogenesis and DNA damage response). These findings align with and mechanistically extend prior functional genomics studies [26,27], demonstrating that collagen-bound DDR1 orchestrates stromal crosstalk, activates PI3K/MAPK cascades, and enables invasive and angiogenic phenotypes across diverse tumors.

Pan-cancer analysis reveals that the association between *DDR1* expression and phenotypic outcomes is divergent and context-dependent (Figure 5C). Pathways exhibiting positive correlation with *DDR1* are predominantly those reliant on ECM remodeling and cell–matrix crosstalk. For instance, angiogenesis is consistently elevated in five stroma-rich solid tumors—retinoblastoma (RB), prostate adenocarcinoma (PRAD), OV, colorectal cancer (CRC), and high-grade glioma (HGG)—suggesting that DDR1 may facilitate neovascularization by aligning tumor collagen fibers to guide endothelial cell invasion. Similarly, EMT and stemness programs are positively associated with DDR1 in renal cell carcinoma (RCC), RB, and glioma, supporting the hypothesis that DDR1-mediated collagen signaling fosters cellular plasticity and therapy resistance.

In contrast, pathways negatively correlated with *DDR1* are frequently related to genome integrity and apoptotic regulation. In uveal melanoma (UVM), RB, and LUAD, high *DDR1* expression is associated with reduced apoptotic signaling (r = −0.33) and suppressed DNA damage and repair responses (r = −0.43 and −0.49, respectively). These consistent negative associations imply that DDR1 may interfere with cell-intrinsic death pathways and impair DNA repair capacity, which could facilitate genomic instability while allowing tumor cells to evade cytotoxic stress [28].

Together, these findings indicate that DDR1 promotes aggressive tumor traits such as angiogenesis, EMT, and stemness in a cancer type–specific manner, while simultaneously downregulating cell death and DNA repair pathways in select tumor contexts. This dual role underscores DDR1’s complexity as both a structural and signaling hub in the tumor microenvironment, with implications for therapeutic targeting.

### 2.4. DDR1 Is a Master Regulator of Immunosuppressive Microenvironments

**Figure 6:**
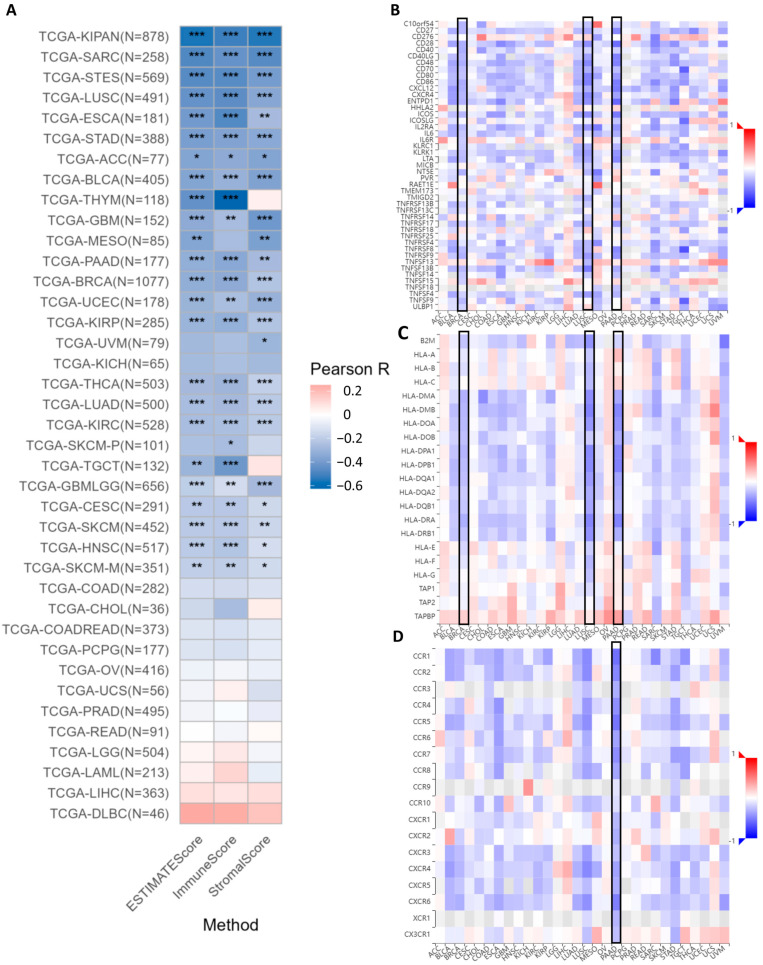
Correlation between *DDR1* expression and immune infiltration across multiple cancer types. (**A**) Negative correlation between *DDR1* expression and immune, stromal, and ESTIMATE scores in multiple cancer types, assessed by Pearson’s correlation (* *p* < 0.05; ** *p* < 0.01; *** *p* < 0.001). (**B**) Spearman correlations between expression of *DDR1* and immunostimulators (Y axis) across human cancers (X axis). Black boxes highlight BRCA, LUSC, and PAAD cancer types. (**C**) Spearman correlations between expression of *DDR1* and MHCs (Y axis) across human cancers (X axis). Black boxes highlight BRCA, LUSC, and PAAD cancer types. (**D**) Spearman correlations between expression of *DDR1* and receptors (Y axis) across human cancers (X axis). A black box highlights the PAAD cancer type.

The analysis of ESTIMATE scores indicates that *DDR1* expression is significantly and negatively correlated with both immune infiltration and stromal components within the tumor microenvironment across multiple cancer types such as PAAD (Figure 6A). DDR1 expression shows significant negative correlations with patient immune infiltrations, as measured by both ESTIMATE scores and immune scores (Supplementary Figures S3 and S4). This suggests that higher *DDR1* expression is associated with a reduced presence of immune cells in the tumor microenvironment. Similarly, the expression of *DDR1* is significantly negatively correlated with the tumor stromal score in 22 cancer types, including IBC (BRCA in TCGA), LUAD, STAD, and PAAD (Supplementary Figure S5).

The online analysis conducted using the GSCA database reveals a robust inverse relationship between *DDR1* expression and key immunomodulatory markers across several cancer types, notably IBC (BRCA in TCGA), LUSC, and PAAD (highlighted by black boxes). In PAAD, *DDR1* expression is significantly inversely correlated with a range of immunostimulatory molecules. These include co-stimulatory markers such as *CD28*, *CD40LG*, *CD48*, *CD70*, *CD86*, and *CD80* (Figure 6B). *DDR1* expression shows a significant negative correlation with multiple major histocompatibility complex (MHC) class II genes, especially in LUSC and PAAD, as depicted in Figure 6C, which implies that *DDR1* may impair antigen presentation [29]. Furthermore, pan-cancer analysis demonstrated a significant inverse correlation between the expression of *DDR1* and immune receptors across multiple malignancies (Figure 6D), with PAAD exhibiting the most pronounced downregulation of immune receptors, as visualized by the deepest blue coloration in the heatmap. Subsequently, we downloaded the uniformly processed pan-cancer dataset (TCGA TARGET GTEx, PANCAN, *n* = 19,131, G = 60,499) from the UCSC database and performed correlation analysis between *DDR1* expression and 60 immune checkpoint pathway markers proposed by Thorsson et al. [30], with results visualized in Supplementary Figure S6.

### 2.5. DDR1-Dependent Regulation of the PDAC Immune Microenvironment

**Figure 7:**
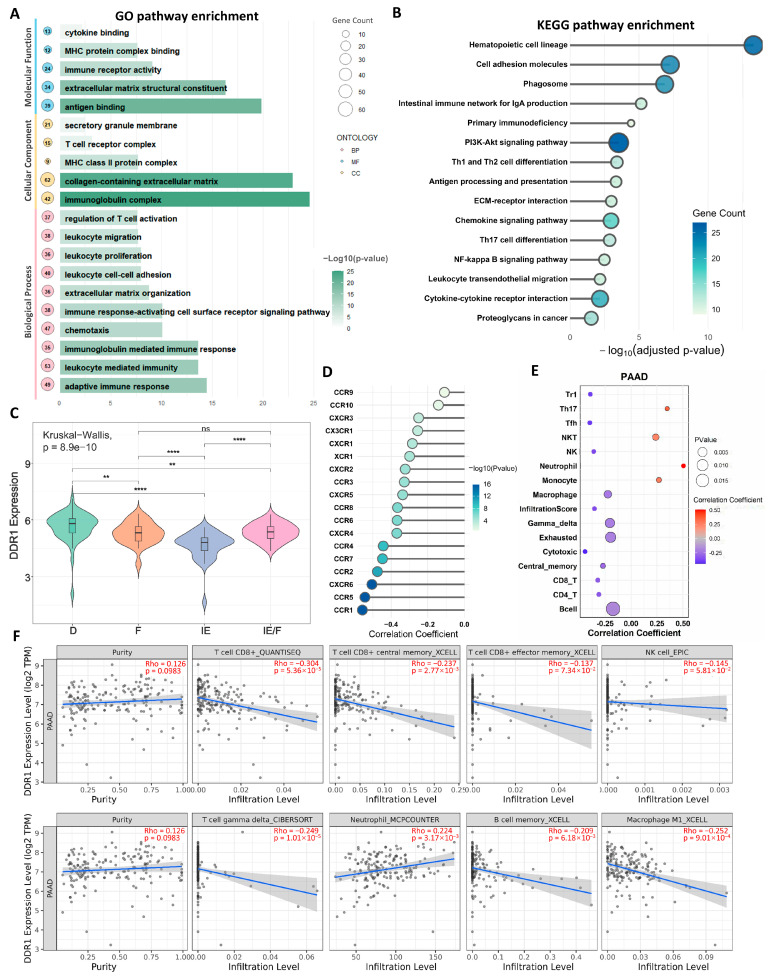
*DDR1* expression is associated with an immunosuppressive tumor microenvironment (TME) in pancreatic ductal adenocarcinoma (PDAC). (**A**) Gene Ontology (GO) enrichment analysis of *DDR1*-correlated genes in PDAC. (**B**) KEGG pathway enrichment analysis of *DDR1*-related genes in PDAC. (**C**) Violin plot showing differential *DDR1* expression across four immune subtypes of the PDAC TME based on the classification by Bagaev et al. [31]: D (desert), F (fibrotic), IE (immune enriched), and IE/F (immune enriched, fibrotic). Kruskal–Wallis test *p*-values are annotated (** *p*  <  0.01; **** *p*  <  0.0001; ns: not significant). (**D**) Correlation analysis between *DDR1* expression and chemokine receptor family genes (*CCR* and *CXCR*) in PDAC. (**E**) Pearson correlation analysis (via the GSCA database) illustrating associations between *DDR1* expression and immune cell types in PDAC. (**F**) Correlation between *DDR1* expression and tumor-infiltrating immune cells in PDAC using the TIMER 2.0 database. Each scatter plot shows Spearman’s correlation between *DDR1* expression and infiltration levels of specific immune cell types. Shaded blue regions denote 95% confidence intervals. Rho and *p*-values are shown on each plot.

We subsequently focused on pancreatic ductal adenocarcinoma (PDAC) based on our pan-cancer analyses revealing DDR1’s pronounced impact in this malignancy, where it demonstrated the strongest association with immunosuppressive signatures. GO (Figure 7A) and KEGG (Figure 7B) enrichment analyses of Differentially expressed genes (DEGs) identified through *DDR1*-based stratification demonstrated significant enrichment in immune-related signaling pathways, suggesting DDR1 contributes to PDAC tumor biology via immunomodulatory mechanisms. Building upon the transcriptomic microenvironment classification proposed by George et al. [31] for PDAC, which identifies four distinct TME subtypes—immune enriched (IE), immune enriched fibrotic (IE/F), fibrotic (F), and immune depleted (D)—our analysis revealed significant heterogeneity in *DDR1* expression across these subtypes (Kruskal–Wallis test, *p* = 8.9 × 10^−10^). Specifically, *DDR1* expression was highest in the D subtype and lowest in the IE subtype, with intermediate expression observed in IE/F and F subtypes (Figure 7C), suggesting DDR1 may serve as a molecular rheostat regulating the immune–fibrotic axis in PDAC microenvironments. Furthermore, we revealed significant inverse correlations between *DDR1* expression and chemokine receptor families (*CCR/CXCR*) in PDAC (Figure 7D), suggesting the *DDR1*-mediated suppression of chemotactic immune cell recruitment.

Using the GSCA database, we identified significant correlations between *DDR1* expression and immune cell infiltration in PDAC. *DDR1* expression showed positive associations with Th17 cells, NKT cells, neutrophils, and monocytes, but negative associations with CD4^+^ T cells, central memory T cells, cytotoxic T cells, γδ T cells, NK cells, and Tfh cells (Figure 7E). Complementary analysis via the TIME 2.0 database confirmed *DDR1*’s negative correlations with CD8^+^ T cells, CD8^+^ central memory T cells, γδ T cells, M1 macrophages, memory B cells, and NK cells in PAAD (Figure 7F).

**Figure 8:**
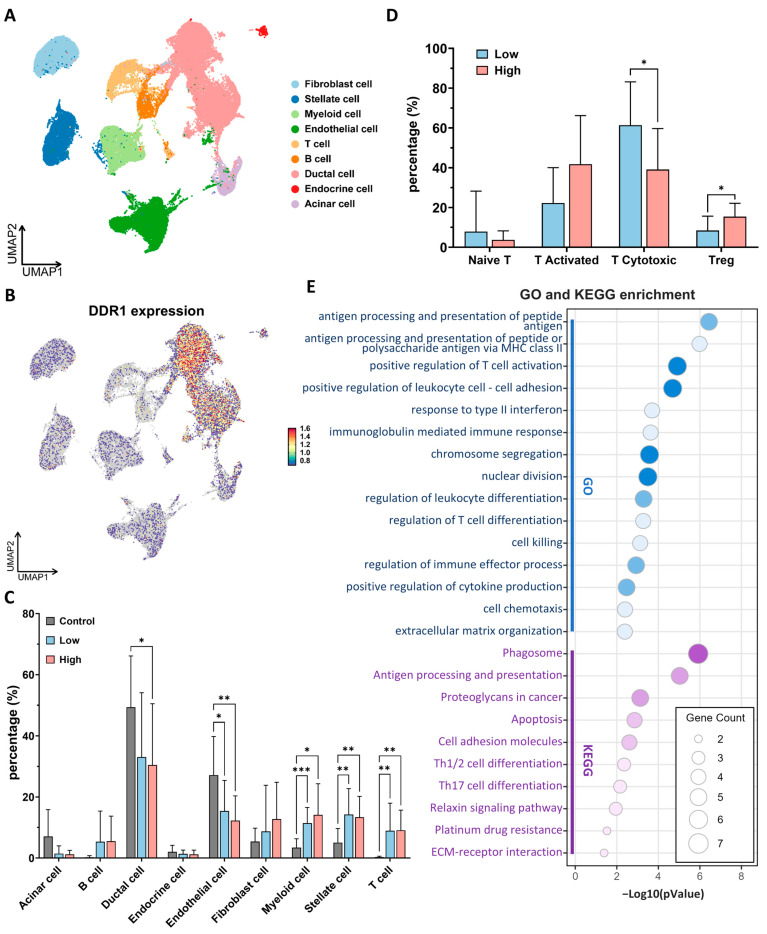
Single-cell sequencing analysis reveals *DDR1*-associated remodeling of the immune landscape in pancreatic ductal adenocarcinoma (PDAC). (**A**) UMAP projection of 24 primary PDAC tumors and 11 normal pancreatic samples, colored by the eight major lineages identified. (**B**) Same UMAP projection colored by normalized *DDR1* transcript abundance. (**C**) Mean ± SD proportion of each lineage in normal pancreas (gray), *DDR1*-low PDAC (blue), and *DDR1*-high PDAC (red) tissues; * *p* < 0.05, ** *p* < 0.01, *** *p* < 0.001 by two-way ANOVA. (**D**) Relative abundance of T cell sub-clusters in *DDR1*-low versus *DDR1*-high PDAC tumors. * *p* < 0.05 by two-way ANOVA. (**E**) GO (blue) and KEGG (purple) over-representation analysis of genes positively correlated with *DDR1* within the cytotoxic T cell cluster.

scRNA-seq profiling of 24 primary PDAC specimens and 11 normal pancreatic tissues (over 50,000 individual cells) revealed that *DDR1* transcripts are almost exclusively confined to ductal-epithelial clusters, with only low-level expression detected in acinar, endocrine or immune/stromal lineages (Figure 8A,B and Supplementary Figures S7 and S8). On this basis we stratified tumors into *DDR1*-high and *DDR1*-low groups according to the median ductal cell signal. Globally, PDAC harbored a markedly remodeled cellular composition relative to a normal pancreas, characterized by an expansion of myeloid, T cell, and stellate fractions, and a concomitant loss of endothelial cells (Figure 8C and Supplementary Figure S10). These broad shifts were comparable in *DDR1*-high and *DDR1*-low tumors; however, a greater granular interrogation of the T cell compartment uncovered a striking imbalance: *DDR1*-high tumors were depleted of cytotoxic CD8^+^ T cells and enriched for Tregs (Figure 8D and Supplementary Figure S9). An analogous inverse relationship between *DDR1* and CD8^+^ infiltration has recently been reported in TNBC [10], suggesting a conserved mechanism of immune exclusion.

Differential gene expression within cytotoxic T cells from *DDR1*-high versus *DDR1*-low tumors followed by GO/KEGG enrichment pinpointed pathways governing antigen processing and presentation, type-I interferon response, leukocyte adhesion, and effector-cell killing (Figure 8E). Collectively, these data indicate that elevated epithelial *DDR1* is accompanied by a quantitative loss and functional impairment of tumor-infiltrating cytotoxic T cells, thereby supporting a model in which DDR1-driven matrix remodeling contributes to immune evasion in PDAC.

### 2.6. DDR1 Antibody Demonstrated Significant Antitumor Effects in PDAC Model

**Figure 9:**
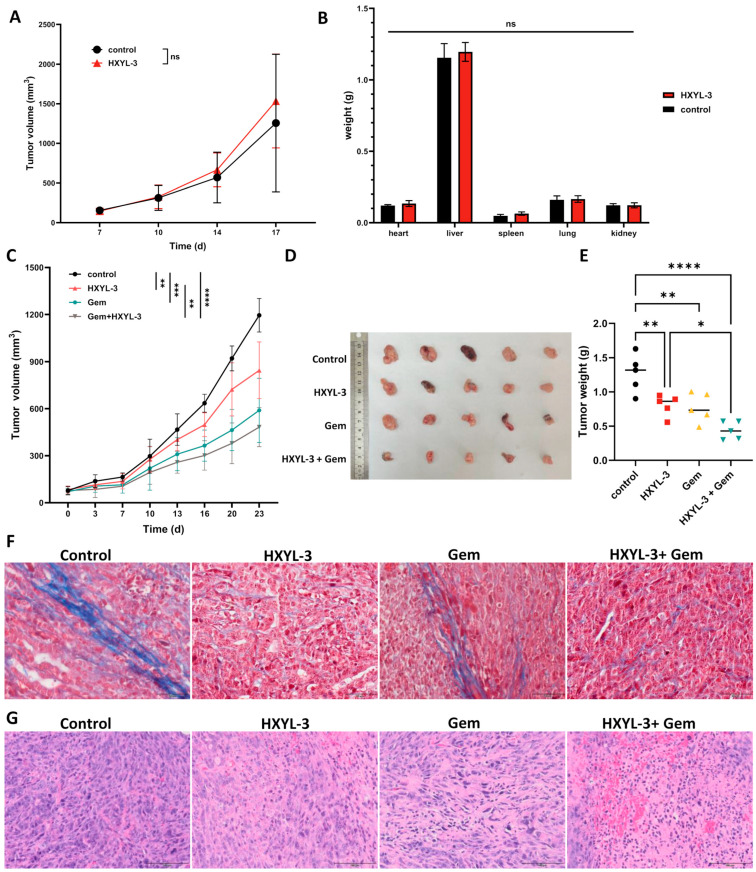
Therapeutic efficacy of DDR1 blockade in pancreatic ductal adenocarcinoma (PDAC) models. (**A**) Tumor growth curves of NSG mice bearing subcutaneous KPC PDAC tumors treated with the DDR1-neutralizing antibody HXYL-3. (**B**) Weight of major organs in HXYL-3-treated NSG PDAC tumor-bearing mice. (**C**–**G**) Combination therapy (Gemcitabine + HXYL-3) in immunocompetent *C57BL/6J* KPC PDAC model: (**C**) Tumor growth curves. (**D**) Endpoint tumor morphology. (**E**) Tumor weight quantification. (**F**) Masson trichrome staining of tumor tissues. Scale bar = 100 μm. (**G**) H&E staining of tumor tissues. Scale bar = 100 μm. Data are presented as mean  ±  SD (n  =  5 per group). Tumor growth curves were analyzed by two-way repeated-measures ANOVA with Holm–Šídák correction (** *p* < 0.01; *** *p* < 0.001; **** *p* < 0.0001; ns: not significant); endpoint comparisons were performed using one-way ANOVA with Tukey’s post hoc test (* *p* < 0.05; ** *p* < 0.01; **** *p* < 0.0001). Analyses were performed in GraphPad Prism 10.3.0.

Motivated by compelling evidence of *DDR1*’s role in shaping immunosuppressive PDAC microenvironments, we therapeutically targeted DDR1 using a novel neutralizing antibody (HYXL-3) to assess blockade efficacy in reversing immune evasion and inhibiting tumor progression. In severely immunodeficient NSG mice bearing subcutaneous KPC PDAC tumors, treatment with the DDR1-neutralizing antibody alone did not significantly inhibit tumor growth compared to human IgG1-Fc controls (Figure 9A). Recognizing that gemcitabine is the current standard-of-care chemotherapeutic agent for PDAC [32], and that its efficacy is often limited by dense stromal barriers and poor immune infiltration [33], we incorporated gemcitabine in combination studies to test whether DDR1 blockade could enhance drug delivery and antitumor immune responses. This strategy aimed to assess the potential synergistic benefit of combining immune-permissive stromal remodeling via DDR1 inhibition with cytotoxic chemotherapy. In immunocompetent *C57BL/6J* mice bearing KPC PDAC tumors, HXYL-3 monotherapy significantly reduced tumor burden, while the combination of HXYL-3 with gemcitabine (Gem) produced the most pronounced tumor suppression (Figure 9C–E), indicating that DDR1 blockade enhances the therapeutic effect of chemotherapy through immune-mediated mechanisms.

Masson’s trichrome staining was performed on murine tumor tissues to evaluate collagen architecture, with collagen fibers staining blue and cytoplasm red. As depicted in Figure 9F, tumors from the control and Gem-treated groups exhibited well-organized collagen fiber alignment, whereas those from the DDR1-neutralizing antibody (HXYL-3) and combination (HXYL-3 + Gem) groups demonstrated markedly disrupted and disorganized collagen structures. These observations indicate that anti-DDR1 treatment significantly impairs the orderly arrangement of collagen fibers within the tumor stroma, potentially facilitating enhanced immune cell infiltration and antitumor immune responses. Additionally, hematoxylin and eosin (H&E) staining was conducted to assess histopathological changes in the tumor tissues (Figure 9G). Tumors from the HXYL-3-treated group displayed a modest reduction in cellular density, along with moderate necrosis and inflammatory responses, as evidenced by focal perivascular inflammatory cell infiltration. And Gem treatment caused nuclear fragmentation with apoptotic features. The combination treatment group (HXYL-3 + Gem) exhibited extensive necrotic regions with pronounced inflammatory infiltration, indicative of an enhanced antitumor immune response.

**Figure 10:**
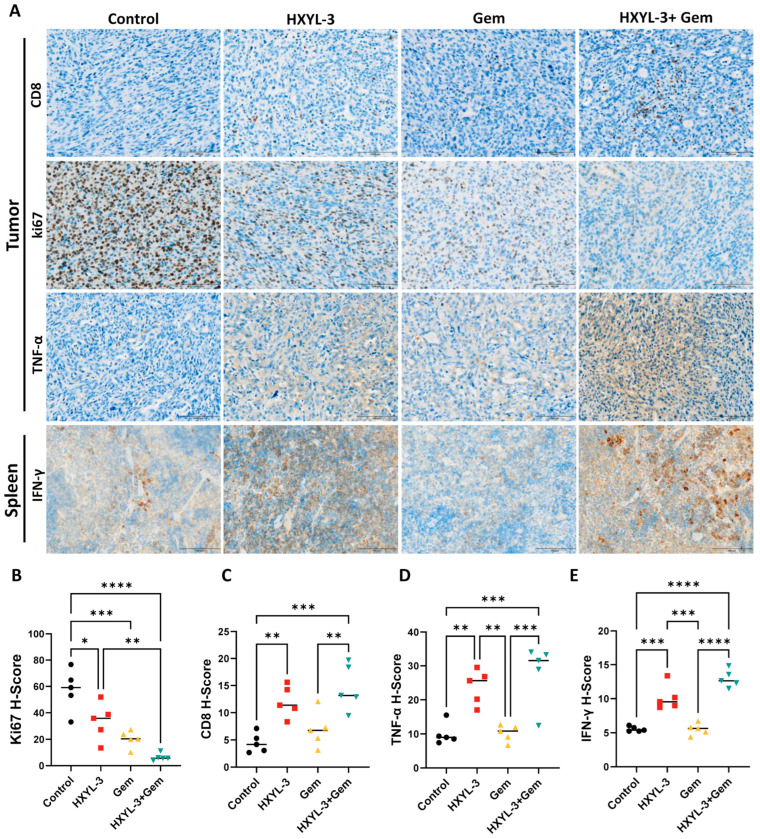
Representative immunohistochemistry and quantification of Ki67, CD8, TNF-α, and IFN-γ in treated *C57BL/6J* KPC PDAC mice. (**A**) Representative immunohistochemical staining images of CD8, Ki67, and TNF-α in tumor tissues and IFN-γ in spleen tissues. Scale bar = 100 μm. (**B**) H-score quantification of Ki67 expression in tumor tissues. (**C**) H-score quantification of CD8 expression in tumor tissues. (**D**) H-score quantification of TNF-α expression in tumor tissues. (**E**) H-score quantification of IFN-γ expression in spleen tissues. Data are presented as mean ± SD (n = 5 per group). Statistical comparisons were performed using one-way ANOVA followed by Tukey’s post hoc test (* *p* < 0.05; ** *p* < 0.01; *** *p* < 0.001; **** *p* < 0.0001). Analyses were conducted using GraphPad Prism (version 10.3.0).

Consistent with a heightened immune response, both HXYL-3 monotherapy and the HXYL-3 + Gem combination produced a marked increase in intratumoral CD8^+^ T cell infiltration (Figure 10A,C) together with higher intratumoral TNF-α levels (Figure 10A,D) and elevated splenic IFN-γ concentrations (Figure 10A,E). The combination treatment further reduced the proportion of Ki67-positive proliferating tumor cells (Figure 10A,B), indicating strong antiproliferative activity. Together with the extensive tumor necrosis and pronounced inflammatory infiltrates, these findings show that the combined inhibition of DDR1 and chemotherapy produces the most potent antitumor effect, likely through an augmented immune-mediated response.

Notably, HXYL-3 caused no major organ weight changes in NSG mice (Figure 9B) or histopathological toxicity in major organs (heart, liver, spleen, lung, and kidney; see Supplementary Figure S11), confirming its safety profile. And the combination regimen did not result in increased systemic toxicity compared with the monotherapy groups.

**Figure 11:**
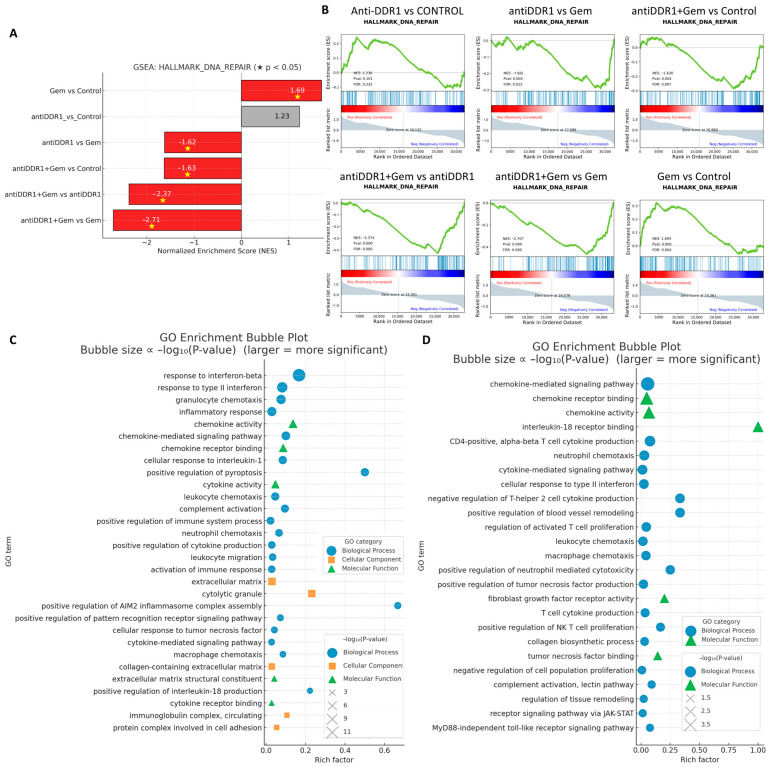
Transcriptomic effects of DDR1 blockade alone and in combination with gemcitabine. (**A**) Hallmark GSEA bar plot for the “DNA Repair” signature across six pairwise comparisons. Bars with red shading and yellow stars indicate statistically significant enrichment (*p* < 0.05). (**B**) Enrichment plots for "HALLMARK_DNA_REPAIR" in the same six contrasts show green curves representing the running enrichment score (ES) profile, blue vertical lines in the middle panel indicating positions of gene set members within the ranked gene list, and bottom heatmaps depicting gene-phenotype correlations with red indicating positive associations and blue indicating negative associations. (**C**) GO bubble plot for genes upregulated by anti-DDR1 monotherapy relative to control. (**D**) GO bubble plot for genes upregulated by anti-DDR1 and gemcitabine combination therapy versus gemcitabine alone.

To clarify the molecular consequences of DDR1 blockade and its interaction with chemotherapy, we performed bulk RNA sequencing on tumors harvested from the four treatment arms and subjected the resulting differential gene lists to Hallmark GSEA and GO interrogation (Figure 11). Hallmark analysis showed that anti-DDR1 monotherapy did not alter the “DNA-repair” signature relative to control, whereas gemcitabine alone produced the expected upregulation of this pathway. Strikingly, addition of HXYL-3 converted the Gem-induced activation into a significant downregulation (Figure 11A,B), implicating DDR1 blockade in the suppression of compensatory repair mechanisms and suggesting a mechanistic basis for the observed synergistic cytotoxicity. These data support the rationale for combining DDR1 inhibition not only with Gem but also with DNA-damage response inhibitors such as PARP or WEE1 antagonists [34]. GO enrichment of genes upregulated by anti-DDR1 monotherapy revealed a coherent induction of innate- and adaptive-immune programs, including type I/II interferon responses, chemokine production, granulocyte and leukocyte chemotaxis, and complement activation (Figure 11C). When HXYL-3 was combined with gemcitabine, the transcriptome shifted even further toward immunostimulation: chemokine-mediated signaling, neutrophil and macrophage chemotaxis, NK- and cytotoxic-T cell activation, MyD88-independent Toll-like-receptor signaling, and JAK/STAT pathways were all preferentially enriched (Figure 11D). Concomitant enrichment of matrix-remodeling terms, such as “collagen biosynthetic process” and “ECM–receptor interaction”, is consistent with the histological evidence that DDR1 blockade disrupts stromal architecture and thereby facilitates immune cell infiltration.

Collectively, these transcriptomic data indicate that DDR1 inhibition dismantles both the physical (collagen alignment) and molecular (DNA repair compensation, immunosuppressive signaling) barriers to therapy, converting gemcitabine-treated tumors into a highly inflamed and immunologically vulnerable state.

## 3. Discussion

This study significantly expands upon previous work by providing a more detailed and comprehensive exploration of *DDR1*’s role in tumor immunity and microenvironment regulation. Although *DDR1* has been previously implicated in PDAC [35,36,37], the current work uniquely integrates extensive bioinformatic analyses and in-depth immune profiling, uncovering detailed associations between *DDR1* expression and specific immune cell infiltration patterns, T cell subpopulation alterations, and broader immune landscape shifts across multiple cancer types.

Our integrative analysis identifies *DDR1* as a critical regulator of the immune-excluded tumor microenvironment across multiple cancers. High *DDR1* expression broadly correlated with reduced immune cell infiltration and lower immune/stromal scores, indicative of an immunosuppressive tumor niche. Notably, tumors with *DDR1* gene amplification displayed among the most immune-excluded phenotypes, suggesting a dose-dependent relationship between *DDR1* expression and immune evasion. Mechanistically, DDR1 appears to enforce immune exclusion through both structural and molecular mechanisms. By interacting with collagen, DDR1 promotes ECM stiffness, forming a physical barrier that limits T cell infiltration. Concurrently, DDR1 signaling activates immunosuppressive pathways such as the CXCL5/NF-κB axis, recruits neutrophils and Tregs [6,38], and triggers PI3K/AKT and JNK cascades that elevate *PD-L1* expression and suppress MHC class II antigen presentation. These combined alterations impair antigen presentation and T cell priming, thereby establishing an immune-excluded environment. Consistently, *DDR1* expression negatively correlates with genes involved in co-stimulatory signaling (e.g., *CD28*, *CD80/86*) and antigen presentation pathways, reinforcing its role in converting tumors into immune deserts.

Our findings align well with and extend recent studies on DDR1-mediated immune exclusion. Sun et al. demonstrated that DDR1-mediated collagen fiber alignment physically restricts cytotoxic T cell infiltration, and *DDR1* genetic ablation in breast cancer models markedly enhances CD8^+^ T cell recruitment. Therapeutically, antibodies targeting DDR1 disrupt collagen organization, facilitating T cell entry and increasing IFN-γ production, leading to potent tumor regression in preclinical models [10]. Functional CRISPR screens also support *DDR1* as a negative regulator of antitumor immunity, revealing that *DDR1* loss increases tumor susceptibility to cytotoxic T lymphocyte and NK cell killing. Additionally, *DDR1* expression positively correlates with immunosuppressive cell populations, such as MDSCs and M2-polarized tumor-associated macrophages (TAMs), highlighting its involvement in fostering an immune-suppressive microenvironment. Collectively, these findings position DDR1 as a master regulator that orchestrates both structural and molecular immune exclusion, making it a compelling therapeutic target.

In PDAC, DDR1’s immunoregulatory role appears particularly pronounced. Our analysis revealed PDAC as among the strongest examples of DDR1-associated immune exclusion, aligning with the tumor type’s known immune-desert phenotype [39]. Transcriptomic profiling of PDAC tumors indicated highest *DDR1* expression within immune-depleted subtypes, inversely correlating with chemokine receptors essential for immune cell trafficking, such as *CCR* and *CXCR* families. scRNA-seq results further confirmed DDR1 expression predominantly in malignant ductal cells, revealing significant differences in immune composition between *DDR1*-high and *DDR1*-low tumors. Specifically, *DDR1*-high tumors showed marked depletion of CD8^+^ cytotoxic T cells and enrichment of immunosuppressive Tregs, consistent with observations in other tumor types such as TNBC [40] and microsatellite-stable colorectal cancer [41]. Furthermore, CD8^+^ T cells infiltrating *DDR1*-high PDAC tumors exhibited transcriptomic signatures indicating functional impairment, including downregulation of pathways involved in antigen presentation, interferon response, and cytotoxic activity.

Additionally, immune profiling highlighted DDR1’s broader impact on immune composition in PDAC, positively associating with immunosuppressive populations such as Th17 cells, neutrophils, NKT cells, and inflammatory monocytes, while negatively correlating with antitumor effectors like conventional CD4^+^ T cells, γδ T cells, NK cells, and M1 macrophages. Notably, *DDR1*-high PDAC tumors skew toward M2-polarized macrophages, a phenotype typically associated with immunosuppression and tumor progression [42]. Previous reports similarly implicate DDR1 in promoting neutrophil extracellular trap (NET) formation [6,43] and suppressing IL-18-driven T cell recruitment [7], reinforcing its role in shaping immune exclusion. Overall, these findings suggest *DDR1*-high PDAC tumors foster an environment unfavorable to antitumor immunity, providing strong rationale for DDR1-targeted therapeutic strategies.

Our in vivo findings underscore that therapeutic targeting of DDR1 can reshape the TME and enhance treatment responses of PDAC, especially when combined with standard chemotherapy. Using an immunocompetent KPC mouse model, we observed that anti-DDR1 antibody treatment significantly disrupted collagen architecture, increased intratumoral CD8^+^ T cell infiltration, elevated local TNF-α, and enhanced IFN-γ production in spleen, indicative of a robust antitumor immune response. Importantly, the antitumor effects of DDR1 blockade depended on host immunity, as evidenced by the negligible impact in immunodeficient NSG mice, highlighting the essential role of immune activation in DDR1-targeted therapies.

Combining the DDR1 blockade with gemcitabine produced striking therapeutic synergy, resulting in maximal tumor growth suppression and pronounced inflammatory infiltration. Tumors receiving combination therapy exhibited extensive necrosis, increased CD8^+^ T cell infiltration, and reduced tumor cell proliferation (Ki-67) compared to monotherapy groups. Transcriptomic analyses revealed that while gemcitabine alone activated DNA repair pathways—a known resistance mechanism [34]—co-treatment with anti-DDR1 antibody substantially suppressed these pathways, suggesting DDR1 is required for optimal activation of DNA damage repair in response to chemotherapy. Although further mechanistic studies are necessary, these data support evaluating DDR1 inhibitors alongside DNA-damage response-targeted therapies.

Moreover, DDR1 inhibition amplified gemcitabine-induced immunomodulatory effects, shifting the TME toward a strongly immunostimulatory state characterized by enhanced chemokine signaling, granulocyte and macrophage chemotaxis, NK cell activation, cytotoxic T cell function, Toll-like receptor signaling, and JAK/STAT pathways. Particularly notable were pathways linked to NK cell activation and macrophage recruitment, implicating these immune subsets in the therapeutic response alongside T cells. The concurrent enrichment of ECM-remodeling pathways further suggests that stromal disruption by DDR1 blockade facilitates immune cell infiltration and tumor clearance.

Despite encouraging findings, our study has several limitations requiring further investigation. First, many of our conclusions rely on correlative analyses from publicly available transcriptomic datasets, which inherently cannot establish causality. Although the data consistently support DDR1’s role in immune regulation, direct mechanistic studies are necessary to validate these associations. Specifically, experiments should confirm whether DDR1 directly suppresses chemokine signaling or DNA damage response pathways (e.g., ATR/Chk1, RAD51). Future studies are needed to explore how DDR1 inhibition affects DNA repair processes and to determine if this pathway can enhance tumor sensitivity to chemotherapy.

Our in vivo experiments also have limitations. Although we demonstrated increased T cell infiltration via immunohistochemistry, the immune profiling was not comprehensive. Flow cytometry or single-cell profiling should be employed in subsequent studies to examine additional immune subsets, including NK cells and macrophages. Given our transcriptomic signatures suggesting NK cell activation and macrophage chemotaxis upon DDR1 blockade, it is crucial to validate these findings experimentally and assess macrophage polarization (M1 vs. M2) and NK cell cytotoxic activity. Clarifying these points would strengthen evidence that DDR1 inhibition broadly reshapes the tumor immune landscape beyond T cell infiltration. Moreover, given our earlier observations linking high *DDR1* expression to poor response to ICB, a logical next step would be to evaluate the therapeutic synergy between DDR1 inhibition and ICB. Specifically, future studies should test the efficacy of combining HYXL-3 with anti–PD-1 and/or anti–CTLA-4 therapies to determine whether DDR1-targeted stromal remodeling can overcome immune exclusion and sensitize “cold” tumors to checkpoint inhibition. Such combination strategies would provide critical insight into the immune-potentiating effects of DDR1 blockade and its potential to convert resistant tumors into immunologically responsive ones. Elucidating how HYXL-3 modulates not only T cell trafficking but also T cell activation and exhaustion status in the presence of ICB will be essential to guide rational therapeutic combinations.

Additionally, our syngeneic subcutaneous PDAC model may not fully capture the complex tumor–stromal interactions characteristic of human PDAC. Orthotopic or spontaneous tumor models, and eventually clinical trials, are needed to validate that DDR1 blockade effectively remodels collagen and enhances immune infiltration within an appropriate physiological context. Encouragingly, early-phase clinical trials of DDR1 inhibitors and humanized anti-DDR1 antibodies are underway [40], and our findings strongly support exploring these agents in combination therapies for immunologically “cold” tumors such as PDAC. Given our observation that *DDR1*-high tumors are resistant to single-agent immune checkpoint blockade, DDR1 expression might serve as a biomarker to identify patients likely to benefit from DDR1-targeted combination regimens. Finally, DDR1’s influence on immune cell polarization warrants deeper mechanistic exploration. Although our analyses associate DDR1 with M2 macrophage polarization and Th17/Treg differentiation, experimental validation is needed. Conditional DDR1 knockout models or co-culture systems could clarify how DDR1 signaling in tumor or stromal cells drives macrophage polarization or modulates cytokine production favoring immunosuppressive T cell subsets. Additionally, dissecting downstream pathways of DDR1 (e.g., NF-κB, STAT3) will illuminate how ECM remodeling translates into altered immune responses. Understanding these detailed molecular mechanisms will inform optimal integration of DDR1 inhibitors into multimodal treatment strategies.

## 4. Materials and Methods

### 4.1. Expression and Mutation Analysis of DDR1 in Pan-Cancer

We utilized several databases and resources to analyze the expression data of *DDR1* in normal and tumor tissues. The GEPIA 2.0 database (http://gepia2.cancer-pku.cn/ (accessed on 13 February 2025)), the TNMplot database (https://tnmplot.com/analysis/ (accessed on 18 February 2025)) [44], and the TIMER 2.0 database (http://timer.cistrome.org/ (accessed on 20 February 2025)) were used to study the expression level of *DDR1* in pan-cancer. The expression levels of *DDR1* were analyzed in various tissues and cells in the GTEx database (https://www.gtexportal.org/ (accessed on 27 May 2025)) and THE HUMAN PROTEIN ATLAS database (https://www.proteinatlas.org/ (accessed on 18 February 2025)). Somatic mutation frequency and genomic information of *DDR1* mutation in cancers were explored with the “cancer types summary and mutations” and “mRNA vs. study” module using the cBioportal (https://www.cbioportal.org/ (accessed on 27 May 2025) database.

### 4.2. Copy Number Variation Analysis

Comprehensive multi-omics data were acquired from two principal resources: (1) The standardized TCGA pan-cancer dataset (PANCAN, Los Angeles, CA, USA, *n* = 10,535 samples, G = 60,499 genes) through UCSC Xena Browser (https://xenabrowser.net/ (accessed on 30 May 2025)), and (2) Level 4 CNV profiles processed by GISTIC2.0 (DOI:10.1186/gb-2011-12-4-r41) from the GDC portal (https://portal.gdc.cancer.gov/ (accessed on 30 May 2025)). ENSG00000204580 (*DDR1*) expression values were extracted from the pan-cancer dataset, with strict sample inclusion criteria retaining only primary tumor tissues and peripheral blood-derived malignancies (“Primary Tumor” and “Primary Blood Derived Cancer—Peripheral Blood”). Expression matrices underwent log2(x + 0.001) transformation to mitigate heteroscedasticity, followed by integration with corresponding CNV profiles using sample-level identifiers. To ensure statistical robustness, cancer types with fewer than three samples were systematically excluded, yielding a final analytical cohort spanning 29 malignancies. Differential expression analysis was performed in R (v4.3.3) across genomic alteration statuses using a tiered statistical approach: (1) Pairwise comparisons between CNV-altered and diploid groups employed unpaired Wilcoxon Rank Sum tests with continuity correction, (2) multi-group analyses (e.g., across amplification, deletion, and diploid states) utilized Kruskal–Wallis rank-sum tests.

### 4.3. Gene Network Association and Functional Profiling

Protein–protein interaction (PPI) networks centered on DDR1 were systematically reconstructed using the STRING database (v12.0; https://string-db.org/ (accessed on 18 March 2025)). High-confidence interactors were subsequently subjected to GO enrichment analysis focusing on BP. For single-cell functional characterization, we leveraged the Cancer Single-cell State Atlas (CancerSEA; http://biocc.hrbmu.edu.cn/CancerSEA/ (accessed on 20 April 2025)) [45], a curated repository encompassing functional annotations for 41,900 single cancer cells across 25 malignancies. This platform enabled systematic evaluation of *DDR1* expression correlations with 14 predefined tumor-related cellular functional states, including proliferation, invasion, and immune evasion.

### 4.4. GO Functions and KEGG Enrichment Analysis

Samples were stratified into high- and low-*DDR1* expression groups using median expression as the cutoff. DEGs between these groups were identified using the DESeq2 package (v1.30.1) with thresholds of |log_2_ fold change| > 1 and an adjusted *p*-value < 0.05. Functional enrichment analysis of DEGs was performed using the ClusterProfiler R package (v4.0.5). GO annotation covered three domains: BP, Molecular Functions (MF), and Cellular Components (CC). Kyoto Encyclopedia of Genes and Genomes (KEGG) pathway enrichment was analyzed in parallel. Significant terms were filtered at *p* < 0.05 with Benjamini–Hochberg correction.

### 4.5. Prognostic Significance Assessment in Pan-Cancer

The prognostic relevance of *DDR1* expression was systematically evaluated using the Kaplan–Meier Plotter platform (http://kmplot.com (accessed on 27 February 2025)) [44], a validated resource for survival analysis across multiple malignancies. For pan-cancer assessment, we selected mRNA expression data with the Affymetrix probe 210749_x_at, focusing on six representative carcinomas: breast carcinoma, lung cancer, pancreatic cancer (TCGA project code PAAD), gastric cancer, colon cancer, and ovarian cancer. Patients were stratified into high- and low-expression cohorts using the platform’s integrated optimal cutoff algorithm, which maximizes survival difference significance through iterative threshold testing. OS analyses were conducted through the platform’s standardized workflow, generating Kaplan–Meier curves with corresponding log-rank test statistics. Prognostic associations were quantified through hazard ratios (HRs) and 95% confidence intervals derived from Cox proportional hazards models.

### 4.6. Immunotherapeutic Response Association Analysis

The therapeutic relevance of *DDR1* expression in immune checkpoint inhibition was investigated through the ROC Plotter platform (https://rocplot.com/ (accessed on 27 February 2025)) using its dedicated immunotherapy module [19]. This analytical framework enabled systematic evaluation of associations between *DDR1* transcript levels and clinical responses to three major ICI classes: anti-PD-1, anti-PD-L1, and anti-CTLA-4 therapies. To further characterize DDR1’s immunomodulatory role, we leveraged two computational modules within the TIDE database (http://tide.dfci.harvard.edu/ (accessed on 16 June 2025)) [46]. First, the Biomarker Evaluation module facilitated comparative assessment of *DDR1*’s predictive performance against established biomarkers through integrated analysis of 33 ICB therapy cohorts. Second, the Regulator Prioritization module was employed to quantify associations between *DDR1* expression patterns and T cell dysfunction metrics across pan-cancer datasets.

### 4.7. Immune Cell Infiltration Analysis

To investigate associations between *DDR1* expression and tumor immune microenvironment composition, we used the TIMER2.0 webserver (http://timer.cistrome.org/ (accessed on 7 February 2025)). This platform integrates six established deconvolution methods—TIMER, CIBERSORT, quanTIseq, xCell, MCP-counter, and EPIC—to comprehensively estimate immune cell infiltration levels across cancer types. The GSCA database (http://bioinfo.life.hust.edu.cn/GSCA/ (accessed on 23 December 2024)), a curated repository of TCGA-derived multi-omics data, was accessed to retrieve immune infiltration metrics. Within the “Immune cell abundance” module, *DDR1* expression profiles and corresponding “Immune infiltration & mRNA expression” datasets were acquired for the specified cancer types. Following data acquisition, paired expression and immune infiltration matrices were subjected to Pearson correlation analysis using R statistical software (v4.3.3). Statistical significance thresholds were defined as absolute correlation coefficients (|r|) > 0.15 with Benjamini–Hochberg adjusted *p*-values < 0.05, ensuring rigorous control for multiple hypothesis testing.

### 4.8. Immunoinfiltration Analysis

For immunoinfiltration evaluation, we utilized the dataset previously employed in CNV analysis. Following quality control measures that excluded samples with zero expression values, all expression values underwent log2(x + 0.001) transformation for normalization. Gene expression profiles were subsequently mapped to standard GeneSymbol identifiers. The ESTIMATE R package (version 1.0.13; https://bioinformatics.mdanderson.org/public-software/estimate/; https://www.nature.com/articles/ncomms3612 (accessed on 21 December 2024)) was systematically applied to compute stromal, immune, and composite ESTIMATE scores for individual tumor samples based on their expression profiles. This analysis yielded immunoinfiltration scores for 10,163 tumor samples across 44 distinct cancer types. Pairwise Pearson’s correlation coefficients between gene expression and immunoinfiltration scores were calculated using the corr.test function in the psych R package (version 2.1.6), with statistical significance determined through Benjamini–Hochberg adjusted *p*-values [47]. To comprehensively investigate immune system interactions, we employed the TISIDB database (http://cis.hku.hk/TISIDB/ (accessed on 15 January 2025)), an integrative platform incorporating multiple immunological data modalities. This resource facilitated systematic examination of pan-cancer associations between *DDR1* expression levels and key immune regulators, including immunoinhibitory molecules, immunostimulatory factors, MHC components, and immune receptor families. All analyses were conducted through the database’s built-in analytical modules following recommended computational workflows.

### 4.9. Cell Line and Mice Model

Male *C57BL/6J* mice (6 weeks old, 20 ± 2 g, SPF grade) and male NSG mice (6 weeks old, 22 ± 2 g, SPF grade) were obtained from Shanghai Sippr-BK Laboratory Animal Co., Ltd. (Shanghai, China) All animals were housed in a specific pathogen-free facility under standard conditions (22 ± 2 °C, 55 ± 10% relative humidity, 12 h light/12 h dark)*,* with ad libitum access to sterile chow and water. Animals were acclimated for 7 days prior to tumor implantation. No animals died prior to the experimental endpoint. At the conclusion of the study, euthanasia was performed using carbon dioxide in accordance with the American Veterinary Medical Association (AVMA) Guidelines for Euthanasia of Animals to minimize pain and distress. All protocols were approved by the Institutional Animal Care and Use Committee of Fudan University School of Pharmacy (approval code: 2022-03-SY-YL-64; approval date: 13 March 2022) and complied with ARRIVE guidelines.

The primary tumor cell line KPC-derived KPC PDAC mouse model [17] was generously provided by Professor Jun Chen (School of Pharmacy, Fudan University). Cells were maintained in DMEM high-glucose medium (Gibco) supplemented with 10% fetal bovine serum (FBS) and 100 U/mL penicillin–streptomycin (Thermo Fisher Scientific, Waltham, MA, USA) under standard culture conditions (37 °C, 5% CO_2_), with subculturing performed during the logarithmic growth phase.

For tumor implantation, both *C57BL/6J* and NSG mice underwent isoflurane anesthesia and had the right dorsal and axillary regions shaved. KPC cells were suspended in sterile PBS (5 × 10^7^ cells/mL, >98% viability by trypan blue exclusion), and 100 μL was injected subcutaneously into the right dorsal flank. Tumor growth was monitored biweekly using digital calipers. Tumor volume was calculated using the modified ellipsoid formula V = 0.5 × L × W^2^, where L and W represent the longest and shortest perpendicular tumor diameters, respectively. Treatments were initiated when the mean tumor volume reached ≥100 mm^3^. Mice meeting this criterion were randomly assigned (Excel = RAND()) to treatment groups (5 mice per group).

Exclusion criteria were pre-specified as follows: failure to engraft (<100 mm^3^ by day 7), body weight loss > 20%, or morbidity requiring humane euthanasia. One C57BL6/J mouse was excluded prior to randomization for lack of engraftment; no post hoc data were removed.

Group allocations and randomization were performed by investigator A (unblinded to group), while tumor measurement and data analysis were conducted by investigators B and C, who were blinded to treatment groups. Cage positions and measurement order were randomized weekly. Sample size (n = 5 per group) was based on established methodology in a key reference study of DDR1 function in subcutaneous tumor models [10], in accordance with the 3Rs principle. No additional a priori power calculation was performed.

### 4.10. Treatment of KPC PDAC NSG Subcutaneous Model

Ten NSG mice with established tumors (≥100 mm^3^) were randomly assigned to two groups: (1) human IgG1-Fc (10 mg/kg i.p. biweekly) and (2) DDR1-neutralizing antibody HXYL-3 (10 mg/kg i.p. biweekly). Tumor size and body weight were recorded twice weekly. On day 17, all mice were euthanized, and tumors as well as major organs were excised and weighed for analysis.

### 4.11. Treatment of KPC PDAC C57BL/6J Subcutaneous Model

Twenty tumor-bearing *C57BL/6J* mice were randomly allocated into four groups (n = 5 each): (1) human IgG1-Fc (10 mg/kg i.p. biweekly), (2) gemcitabine (Gem, 25 mg/kg i.p. weekly), (3) HXYL-3 (10 mg/kg i.p. biweekly), and (4) HXYL-3 + Gem combination (HXYL-3 10 mg/kg i.p. biweekly + Gem 50 mg/kg i.p. weekly). Tumor size and body weight were monitored twice weekly. On day 23 after treatment initiation, mice were euthanized, and tumors and major organs were collected. The following endpoints were assessed: tumor weight, collagen architecture (Masson’s trichrome staining), necrosis percentage (H&E staining), Ki67 proliferation index, intratumoral TNF-α, and splenic IFN-γ levels, as well as histopathology of major organs.

### 4.12. Single-Cell RNA Sequencing Data Processing and Immune Landscape Analysis

We analyzed >50,000 cells from 24 primary PDAC tumors and 11 normal pancreata (NGDC, PRJCA001063) with Seurat v5.1.0. After discarding cells expressing <200 genes, genes detected in <3 cells, or cells with >10% mitochondrial RNA, each sample was QC-processed separately, normalized by SCTransform, and batch-corrected with Harmony before UMAP clustering. Immune clusters were sub-selected, re-integrated via canonical correlation analysis, and reclustered with UMAP for higher resolution. Tumor ductal cells were dichotomized into *DDR1*-high and DDR1-low groups (median split); we compared cell-type proportions and T cell functional scores, identified differentially expressed genes with FindMarkers (FDR < 0.05), and subjected them to GO and KEGG enrichment using clusterProfiler.

## 5. Conclusions

Our comprehensive pan-cancer analysis demonstrates that *DDR1* is markedly overexpressed in diverse malignancies and is strongly associated with poor clinical outcomes, immunotherapy resistance, and diminished antitumor immune cell infiltration. DDR1 appears to drive tumor progression and immune evasion through its pivotal roles in extracellular matrix remodeling and modulation of immune signaling pathways. Furthermore, preclinical studies in a pancreatic cancer model reveal that targeting DDR1 not only disrupts collagen architecture but also enhances chemotherapeutic efficacy and promotes immune cell infiltration. Collectively, these findings nominate DDR1 as a promising dual stromal-immune therapeutic target for overcoming microenvironment-driven treatment resistance in collagen-rich tumors.

## Data Availability

All data utilized in this study were obtained from publicly accessible databases. For further data support or additional information, please feel free to contact the authors via email.

## Supplementary Materials

The following supporting information can be downloaded at https://www.mdpi.com/article/10.3390/ijms26167731/s1.
