# Supplementary material for: DDR1 Drives Collagen Remodeling and Immune Exclusion: Pan-Cancer Insights and Therapeutic Targeting in Pancreatic Ductal Adenocarcinoma
Source: Int J Mol Sci. 2025 Aug 10;26(16):7731. doi: 10.3390/ijms26167731 (PMC12386709; doi:10.3390/ijms26167731)
Supplement: Supplementary file 1 [file ijms-26-07731-s001.zip › ijms-3706198-supplementary.pdf]

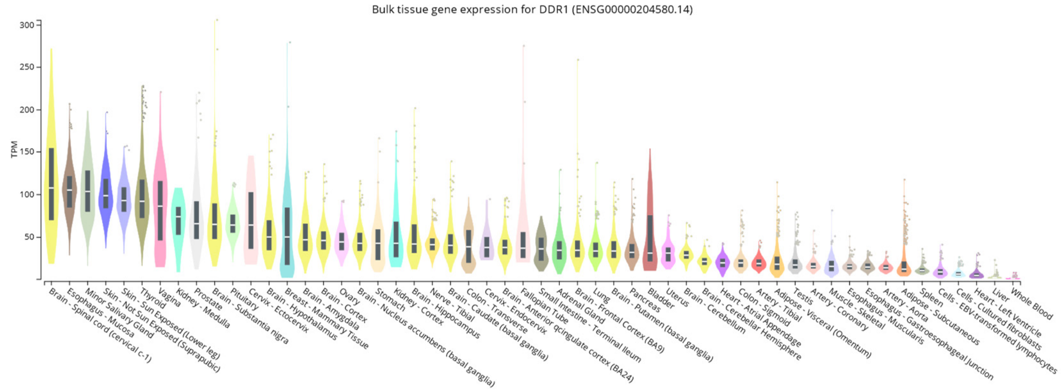

Figure S1. Bulk tissue gene expression for *DDR1* (ENSG00000204580.14) based on the GTEx database.

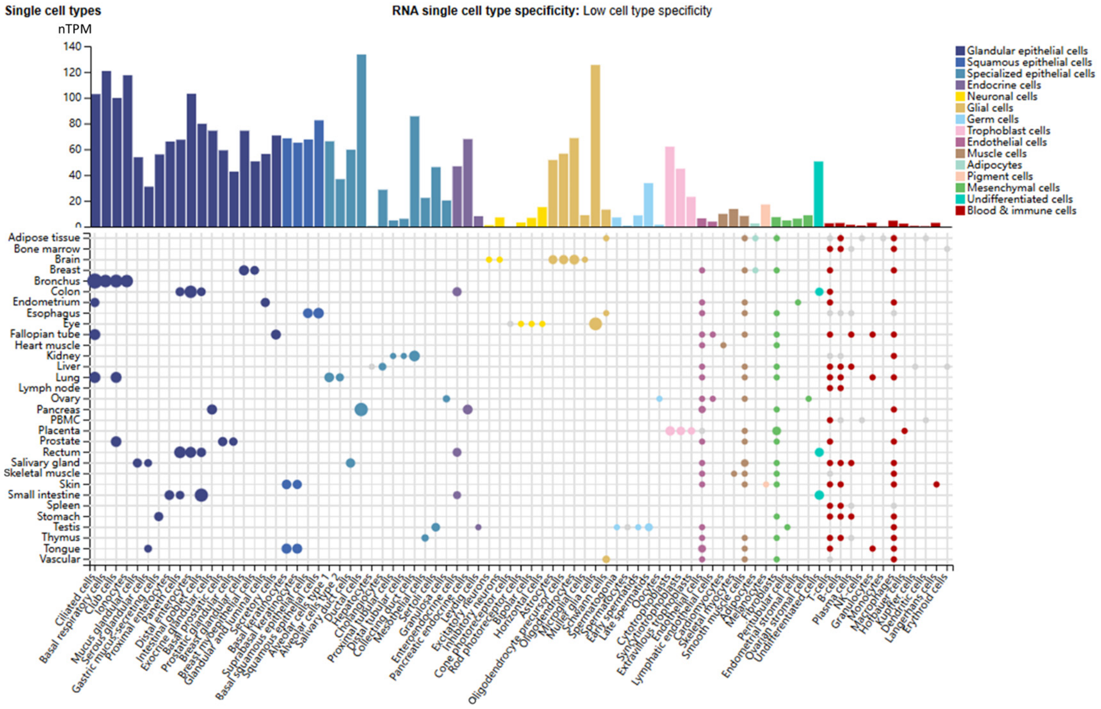

Figure S2. *DDR1* expression in different cells based on the Human Protein Atlas database (v24.0).

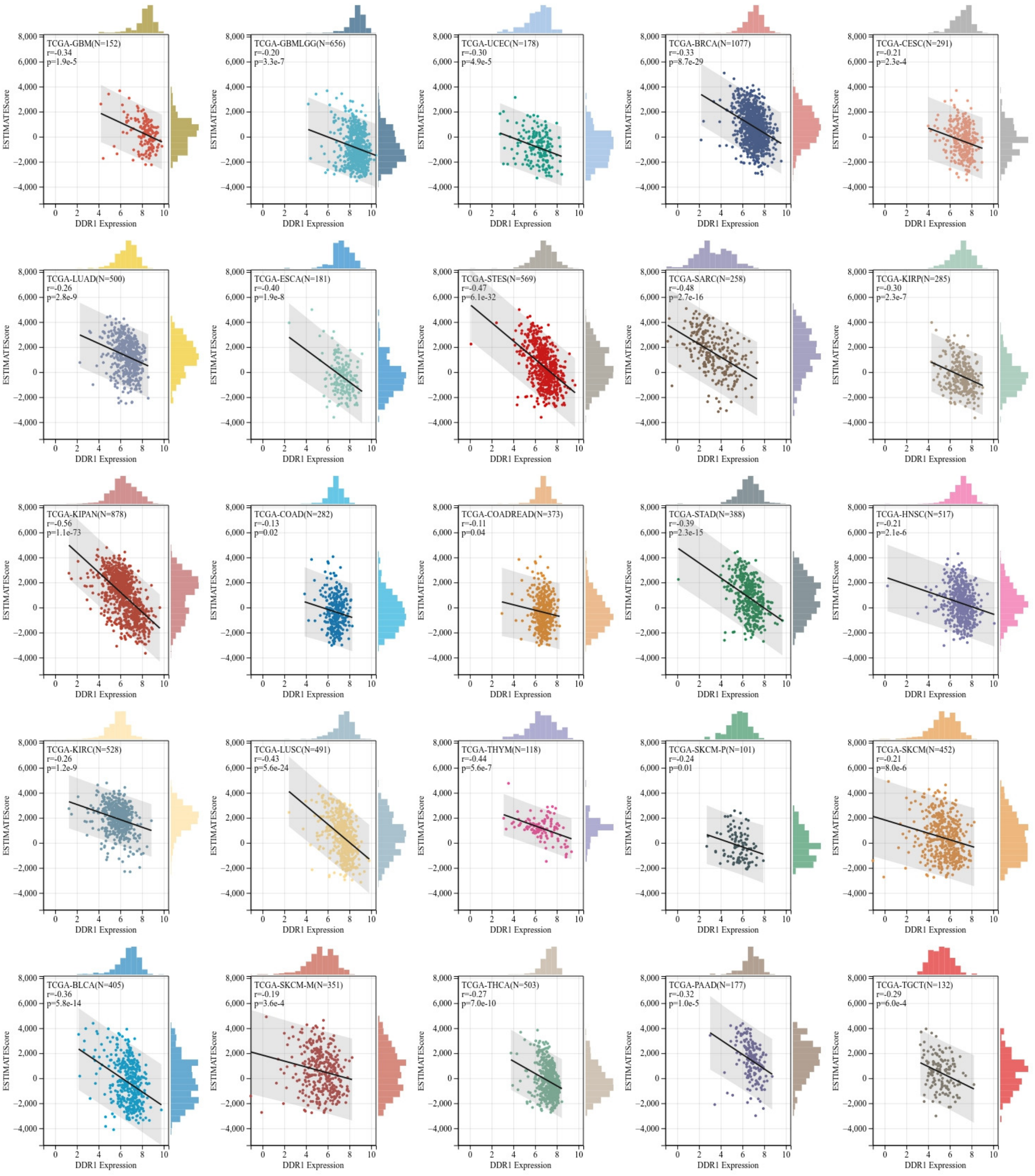

Figure S3. Negative correlation between *DDR1* expression and ESTIMATE scores in 25 cancer types.

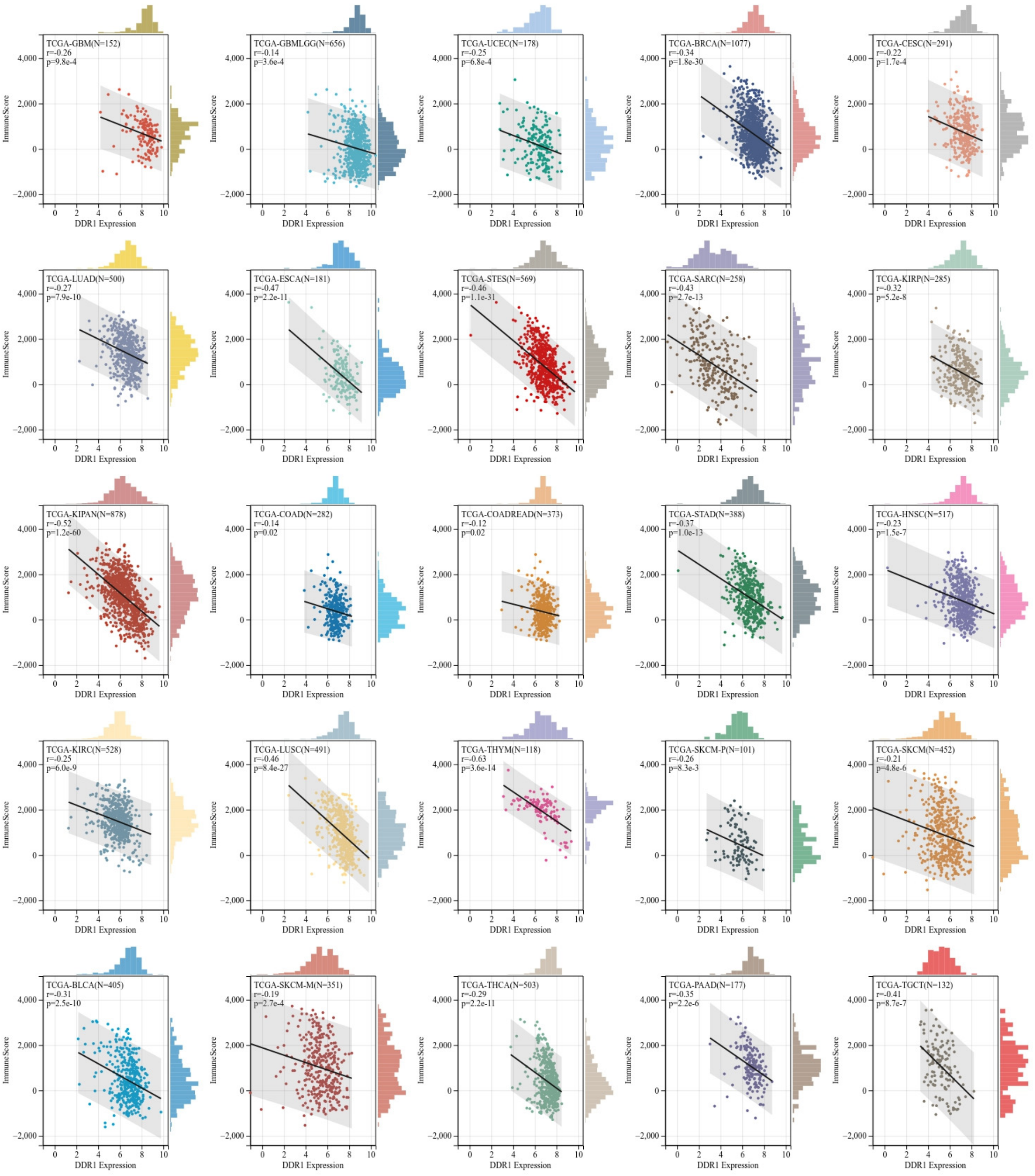

Figure S4. Negative correlation between *DDR1* expression and immune scores in 25 cancer types.

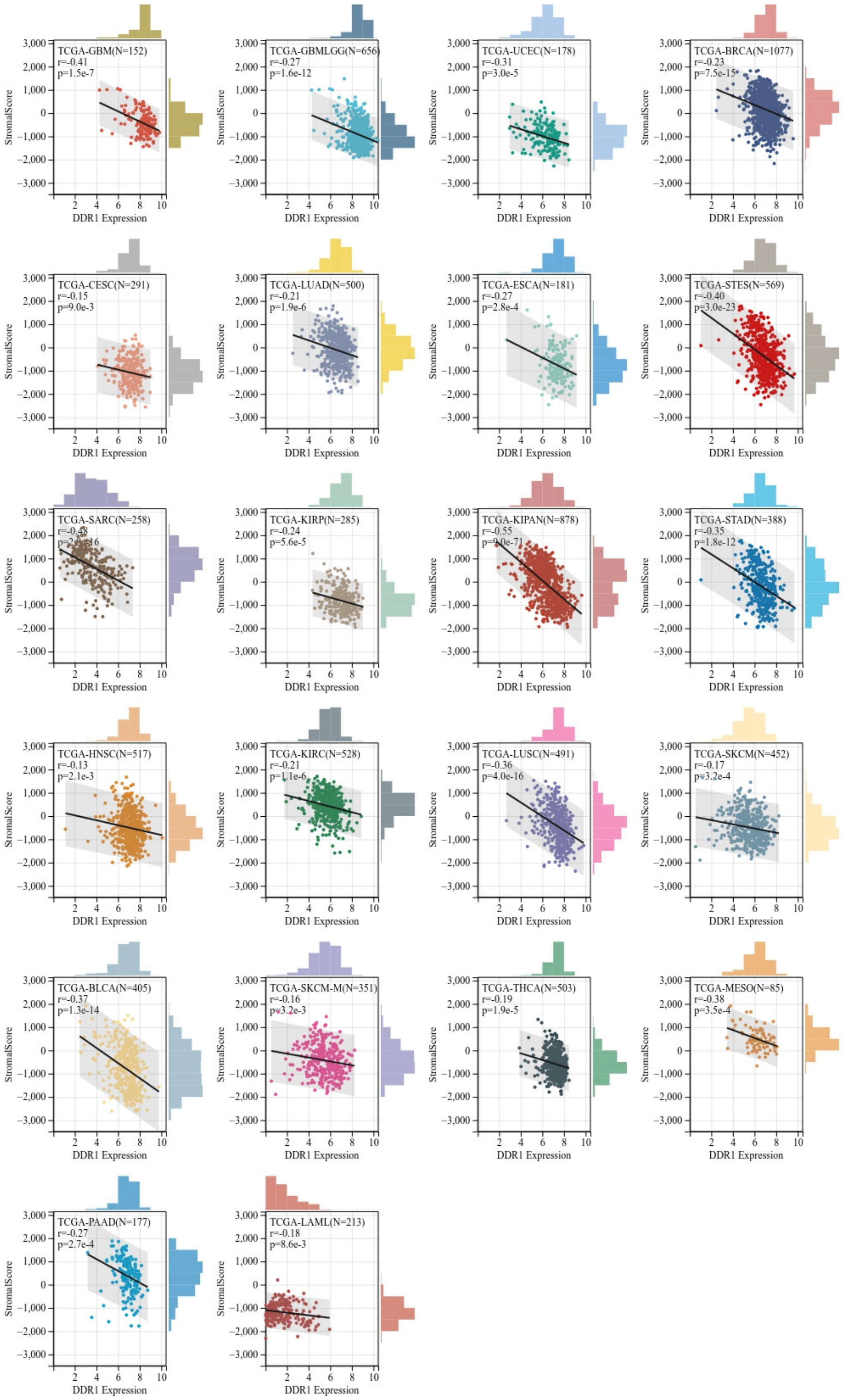

Figure S5. Negative correlation between *DDR1* expression and Stromal scores in 22 cancer types.

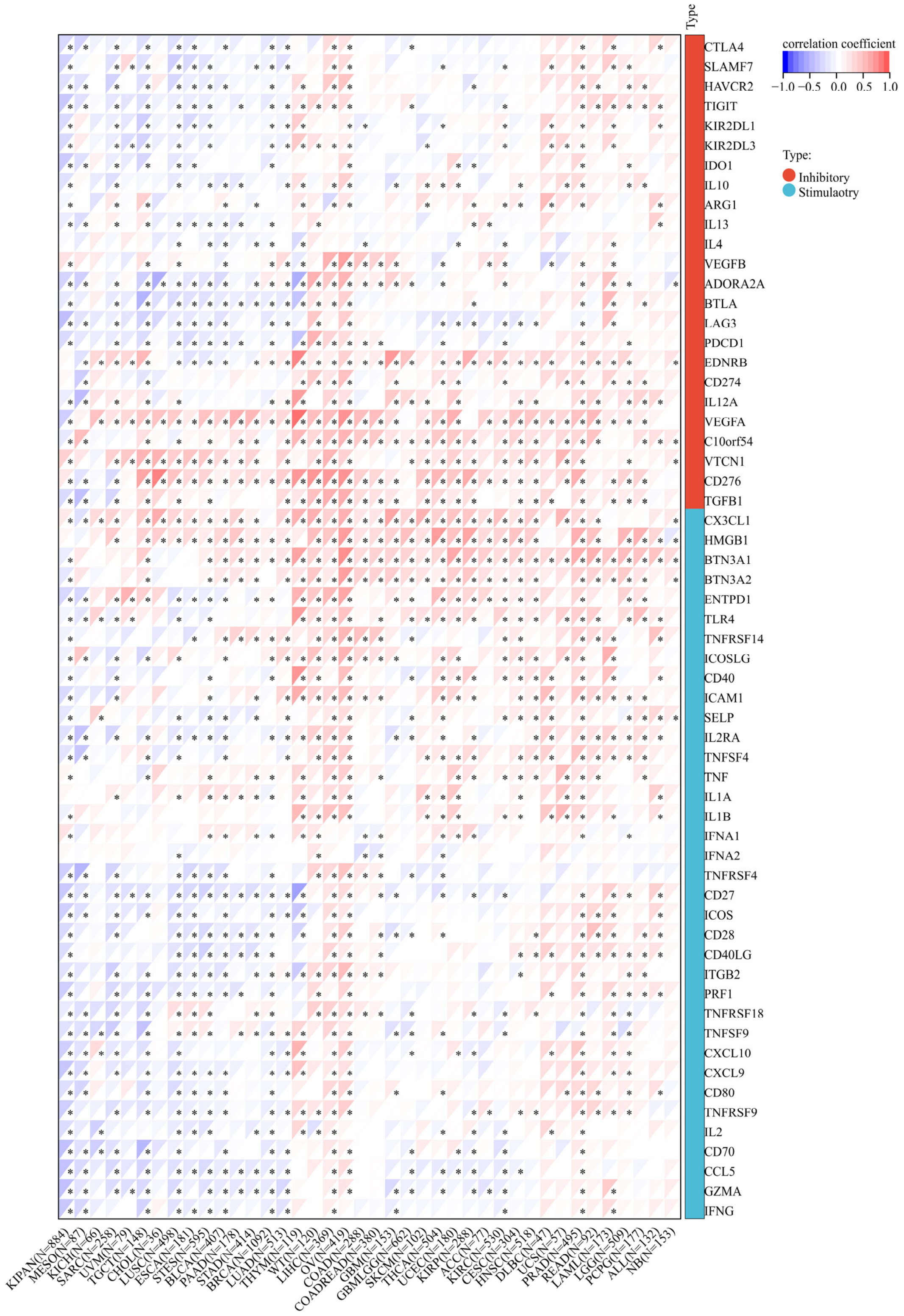

Figure S6. Pan-cancer analysis of correlation between *DDR1* expression and immune checkpoint pathway genes (24 inhibitory, 36 stimulatory), assessed by Pearson's correlation (* $p < 0.05$).

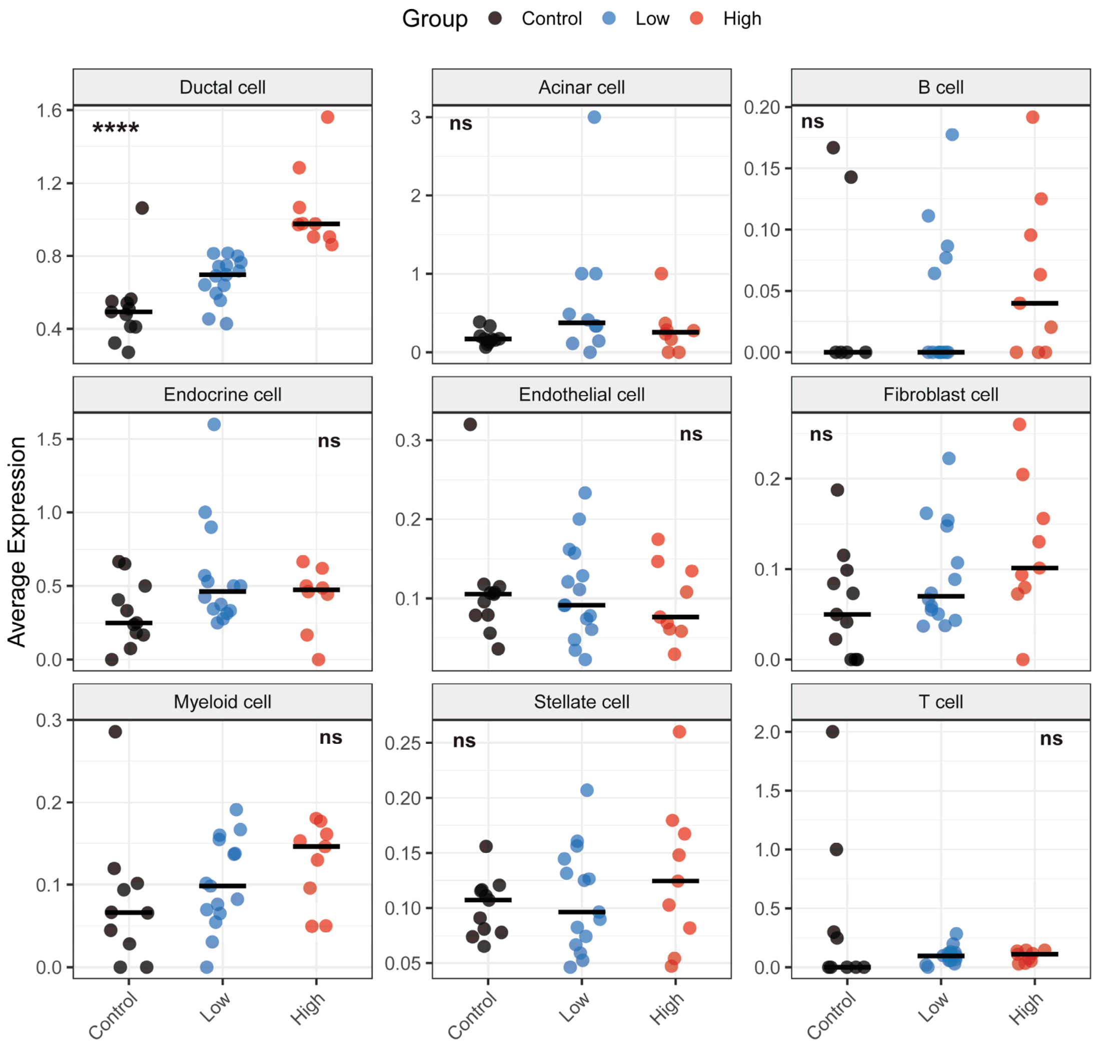

Figure S7. *DDR1* expression in different cell types from 24 primary PDAC tissues (DDR1 high/low) and 11 normal pancreatic tissues (control). Statistical comparisons were performed using one-way ANOVA followed by Tukey's post hoc test (**** $p < 0.0001$; ns: not significant).

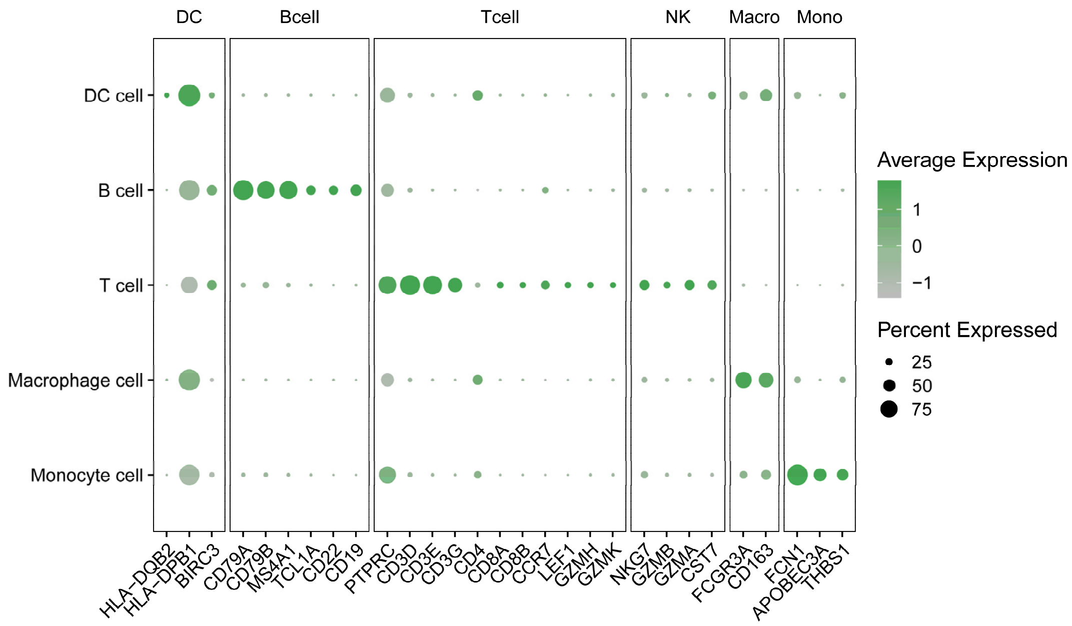

Figure S8. Major immune cell clusters in PDAC, clustered by their relative expression of the cell type-specific markers.

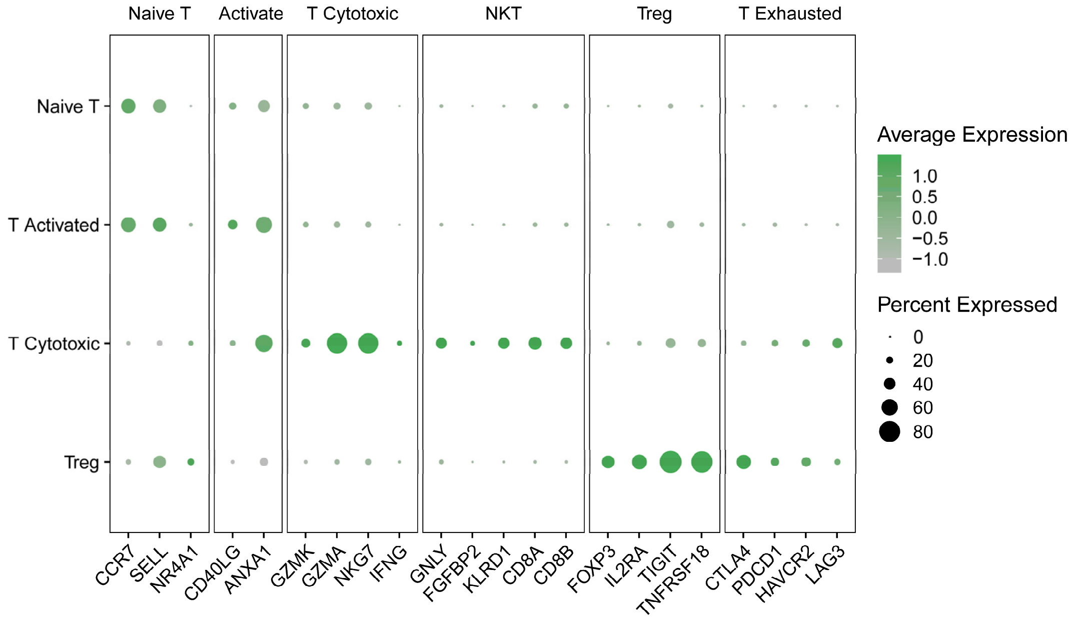

Figure S9. Major T cell subclusters in T cells from PDAC tissue, clustered by their relative expression of the cell type-specific markers.

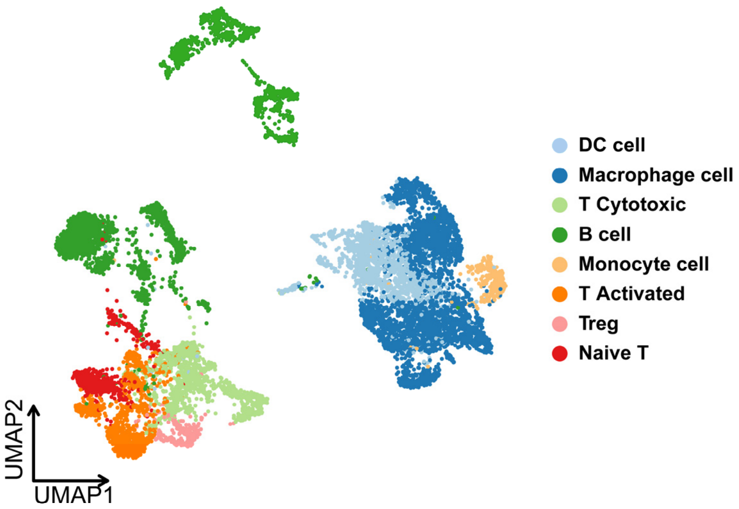

Figure S10. UMAP plot of the identified immune cell types.

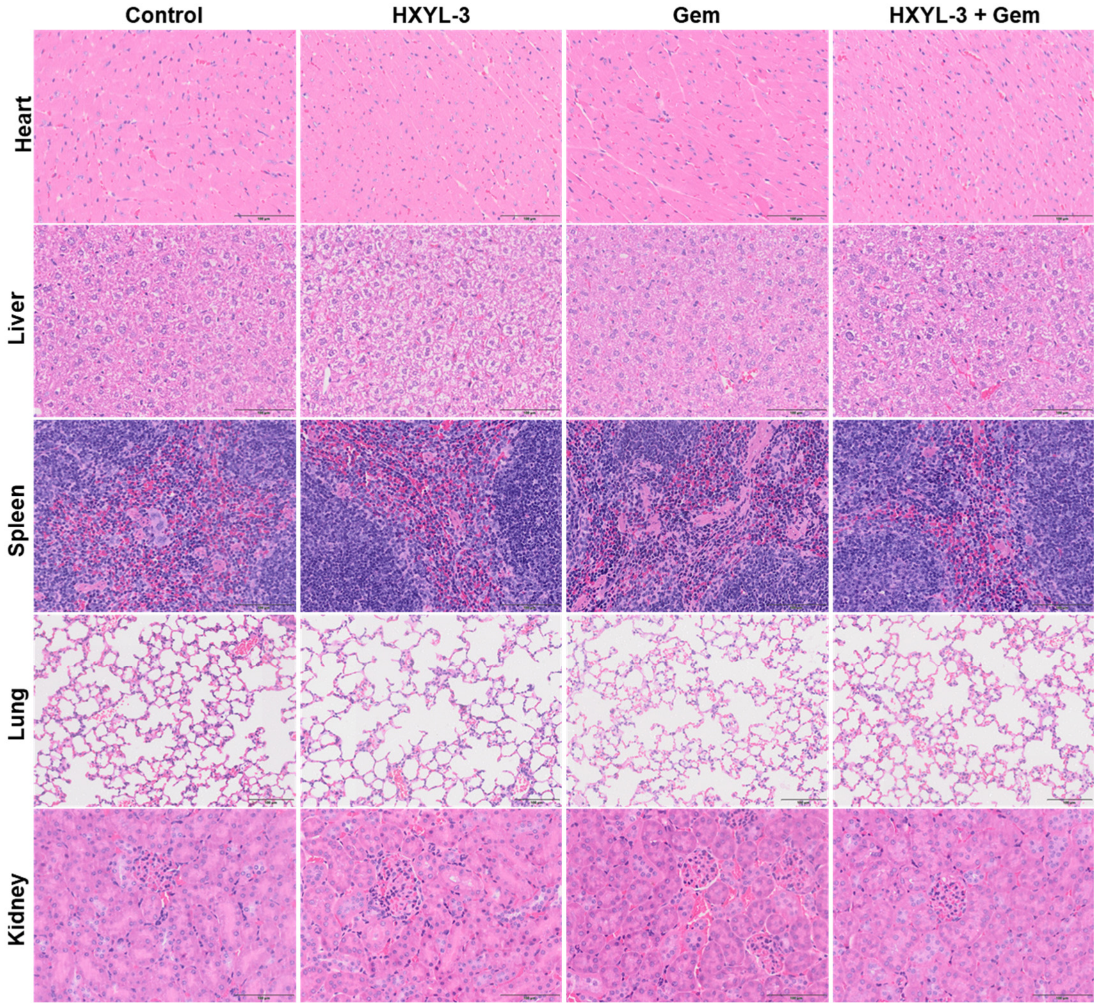

Figure S11. H&E staining results of major organ tissues from *C57BL/6*J KPC pancreatic cancer subcutaneous-tumor-bearing mice after treatment. Scale bar = 100 μm
